# IFI-ML: a novel framework combining Fuzzy Inference and Machine Learning for Parkinson's disease classification

**DOI:** 10.3389/frai.2026.1949960

**Published:** 2026-10-21

**Authors:** Vinothini S., Magesh G.

**Affiliations:** School of Computer Science Engineering and Information Systems, Vellore Institute of Technology, Vellore, TN, India

**Keywords:** clinical decision support, entropy-based feature partitioning, hybrid models, Imbalanced data handling, interpretability in medical AI, machine learning, Parkinson's disease classification

## Abstract

Early detection of Parkinson's Disease (PD) remains challenging due to vocal signal complexity and severe data imbalance, which necessitates that machine learning (ML) models deliver higher classification accuracy. This study aims to break the systematic, inverse trade-off between sensitivity and specificity in voice-based PD classification. We present the Intelligent Fuzzy Inference and Machine Learning (IFI-ML) framework, an uncertainty-aware architecture utilizing entropy-based partitioning, hybrid classifiers, and a Sugeno Type-2 Fuzzy Inference System (FIS). While traditional frameworks apply global resampling techniques such as the Synthetic Minority Over-sampling Technique (SMOTE) or Random Over-Sampling (ROS) across entire datasets, the proposed IFI-ML framework utilizes information gain-driven clustering to segment samples into sensitive (uncertain boundary) and non-sensitive partitions. The framework optimizes conventional ML classifiers on the stable, non-sensitive clusters, while routing critical boundary samples to the Type-2 FIS to handle local class overlap without distorting the overall data distribution. We evaluated our framework on two public, imbalanced datasets from the University of California, Irvine (UCI) Machine Learning repository: Parkinson's voice Dataset 1 (*D*_1_) and Dataset 2 (*D*_2_). The proposed framework achieves an accuracy of 97% on *D*_1_ and 92.17% on *D*_2_, while simultaneously raising specificity to 94-95% on *D*_1_ and 75-88% on *D*_2_. The Matthews Correlation Coefficient (MCC) improves to 0.93 and 0.77, respectively. Furthermore, IFI-ML reduces false-positive and false-negative rates to 3-7% on *D*_1_ across all classifiers, and limits false-positive rates to 11-12% for the K-Nearest Neighbors (KNN) model on *D*_2_. Under a rigorous 10-fold cross-validation (10 CV) scheme, the framework consistently outperforms 15 baseline machine learning models. These benchmark results show the clinical reliability and robustness of the proposed approach, establishing a state-of-the-art benchmark for non-invasive vocal biomarkers.

## 1 Introduction

Parkinson's Disease (PD) is the second most common neurodegenerative disease worldwide affecting over 8.5 million people (Jankovic and Tan, 2020; Poewe et al., 2017). Pathologically, PD is Pathologically, PD is characterized by a chronic, progressive degeneration of substantia nigra pars compacta, and intracellular deposits of misfolded α-synuclein proteins that aggregate to form Lewy bodies (Wang et al., 2026; Ben-Shlomo et al., 2024; Poewe et al., 2017). In clinical practice, early diagnosis is important because symptoms are variable and often overlap with other neurological disorders (Emegano et al., 2026; Oumaima et al., 2025; Emamzadeh and Surguchov, 2018; Rizzo et al., 2016). The clinical course of PD generally ranges from early non-motor vocal dysphonia (speech instability and laryngeal muscle rigidity) to the prominent late motor symptoms such as bradykinesia, resting tremors, and postural instability (Armstrong and Okun, 2020; Memar et al., 2018; Maetzler et al., 2009). In PD clinics, classical diagnostic scales such as the Unified Parkinson's Disease Rating Scale (UPDRS) (Goetz et al., 2008) are not fully reliable, because of subjective evaluation, high dependence on clinician experience and inter-observer variability.

Vocal impairments commonly occur prior to motor symptoms, and therefore, voice-based biomarkers can be an objective, non-invasive and affordable screening alternative for clinical limitations (Tracy et al., 2020; Naranjo et al., 2017; Tsanas et al., 2012). Recent advances have utilized explainable machine learning (ML) methods (e.g., eXtreme Gradient Boosting (XGBoost) and SHapley Additive exPlanations(SHAP)) and acoustic feature enable telemonitoring and classification (Egbo et al., 2025). These datasets are inherently imbalanced (Ge et al., 2023), so researchers often use a global resampling technique such as Synthetic Minority Over-sampling Technique (SMOTE) or Random Over-Sampling (ROS) (Mei et al., 2021; Mahmud et al., 2021).

However, despite these advances, there is a significant “research gap.” In traditional machine learning techniques, there is usually an explicit trade-off between sensitivity and specificity (Tolosa et al., 2021). In many cases, the goal is to maximize recall (sensitivity), while sacrificing specificity. This leads to a large “false positive burden” with unnecessary clinical follow-ups and patient anxiety. In addition, global resampling methods often ignore the boundary uncertainty and therefore yield biased and unstable models for complex and heterogeneous voice data (Sakar et al., 2013; Cigdem and Demirel, 2018). There are currently no frameworks that explicitly consider localized uncertainty instead of global data manipulation (Cao et al., 2024; Shafiq et al., 2023; Goyal et al., 2020; Karapinar Senturk, 2020).

The motivation for Intelligent Fuzzy Inference and Machine Learning (IFI-ML) arises directly from the limitations observed in the comparison above. While existing state-of-the-art (SOTA) models (Zhang et al., 2023; Egbo et al., 2025) provide high global performance, they typically treat the dataset as a monolithic entity. Neither global resampling nor advanced feature engineering (Cigdem and Demirel, 2018) has reliably resolved the inverse correlation between sensitivity and specificity (Tolosa et al., 2021; Salmi et al., 2024). The IFI-ML framework is motivated by the need for an uncertainty-aware architecture that can:

1. Target boundary regions: Use entropy-based Gini partitioning to locate areas where standard ML models are most likely to fail.
2. Maintain the efficiency: improve the classification performance by making use of optimized ML for non-sensitive samples and Type-2 Fuzzy Inference for sensitive ones.
3. Break the trade-off ceiling: To obtain high specificity without sacrificing the sensitivity for early clinical screening.

This is needed to improve clinical reliability since it is better to have a framework that can selectively handle uncertain points without degrading the performance of well-separable cases (Little, 2007; Little et al., 2009; Sakar et al., 2018, 2019). This study is useful as it provides a means to reduce both false negatives (for early neuroprotective care) and false positives (to relieve primary care bottlenecks). In this work, we present the IFI-ML framework with a novel dual-track processing paradigm.

The novelty of the proposed method is in the “uncertainty-aware” architecture which makes use of the entropy-based feature partitioning to divide the data into sensitive and non-sensitive clusters. Unlike existing monolithic models, IFI-ML uses optimized scalable ML models for the rest of the data, and it keeps a Sugeno/Type-2 Fuzzy Inference System (FIS) for important boundary regions where the uncertainty is maximum. This integration enables the framework to overcome the inverse correlation between sensitivity and specificity, and provides a more robust and interpretable tool for early PD detection.

To address these challenges, the present study investigates the following questions:

1. Can entropy-based clustering combined with fuzzy inference improve classification performance on imbalanced Parkinson's voice datasets compared to conventional resampling techniques?
2. Does IFI-ML resolve sensitivity-specificity trade-offs and improve interpretability compared to existing ML models?
3. How does the proposed IFI-ML framework compare to the best methods on different datasets when using different evaluation metrics (accuracy, specificity, MCC)?

## 2 Methods

The proposed research introduces the Intelligent Fuzzy Inference with Machine Learning (IFI-ML) framework, an innovative methodology designed to enhance the early detection and classification of Parkinson's Disease (PD) utilizing voice-based biomarkers. Medical AI has basic trade-off problems that IFI-ML can solve with intelligence and multi-stage processing that goes beyond monolithic models. Using a multi-stage pipeline makes decisions more stable, easier to understand, and better than baseline ML models. The main components of the IFI-ML framework are clustering based on information gain, building hybrid models on clusters, and optimizing Sugeno FIS for sensitive clusters. Our IFI-ML model deals with the variability of Parkinson's by

1. Using an entropy-based feature partitioning method to find groups of samples that are non-sensitive and sensitive.
2. We build the hybrid model with machine learning and FIS to work with both groups, which helps us train the model in a targeted way.
3. This combination of classifiers with FIS makes it easier to understand and stronger on voice biomarkers that aren't balanced;
4. We show through extensive benchmarking how the IFI-ML component affects classification performance both on its own and when combined with other components.

The IFI-ML framework is assessed using a nested double-loop cross-validation protocol to ensure a statistically rigorous and objective evaluation. The outer loop consists of a standard stratified 10-fold cross-validation (10 CV) that splits the dataset into leaf nodes. All pipeline components, including information gain feature scoring, Gini-based tree partitioning, threshold calculation, and classifier training, are fitted solely on the training split dataset *D*. Hyperparameter tuning for the conventional ML classifiers is restricted to an inner 10 CV loop operating strictly within the active training fold. The final performance metrics are evaluated on ML classifiers and FIS in a weighted average method. This guarantees that test samples remain entirely outside both the training and hyperparameter optimization loops, preventing any optimistic performance biases. This architecture also offers a strong, dependable, and deployable framework for classifying Parkinson's disease using voice biomarkers. We will now go over the framework in detail, focusing on Figure 1. After that, we will describe the pseudocode of the IFI-ML Algorithm 1 that shows the whole pipeline and the order of steps. We test the framework on two UCI voice datasets (*D*_1_: 195 samples with 23 features; *D*_2_: 756 samples with 752 features) in Table 1 using 10-fold cross-validation and balancing methods like SMOTE and Random Over-Sampling. These datasets *D*_1_ and *D*_2_ does not have missing values and variables.

**Figure 1:**
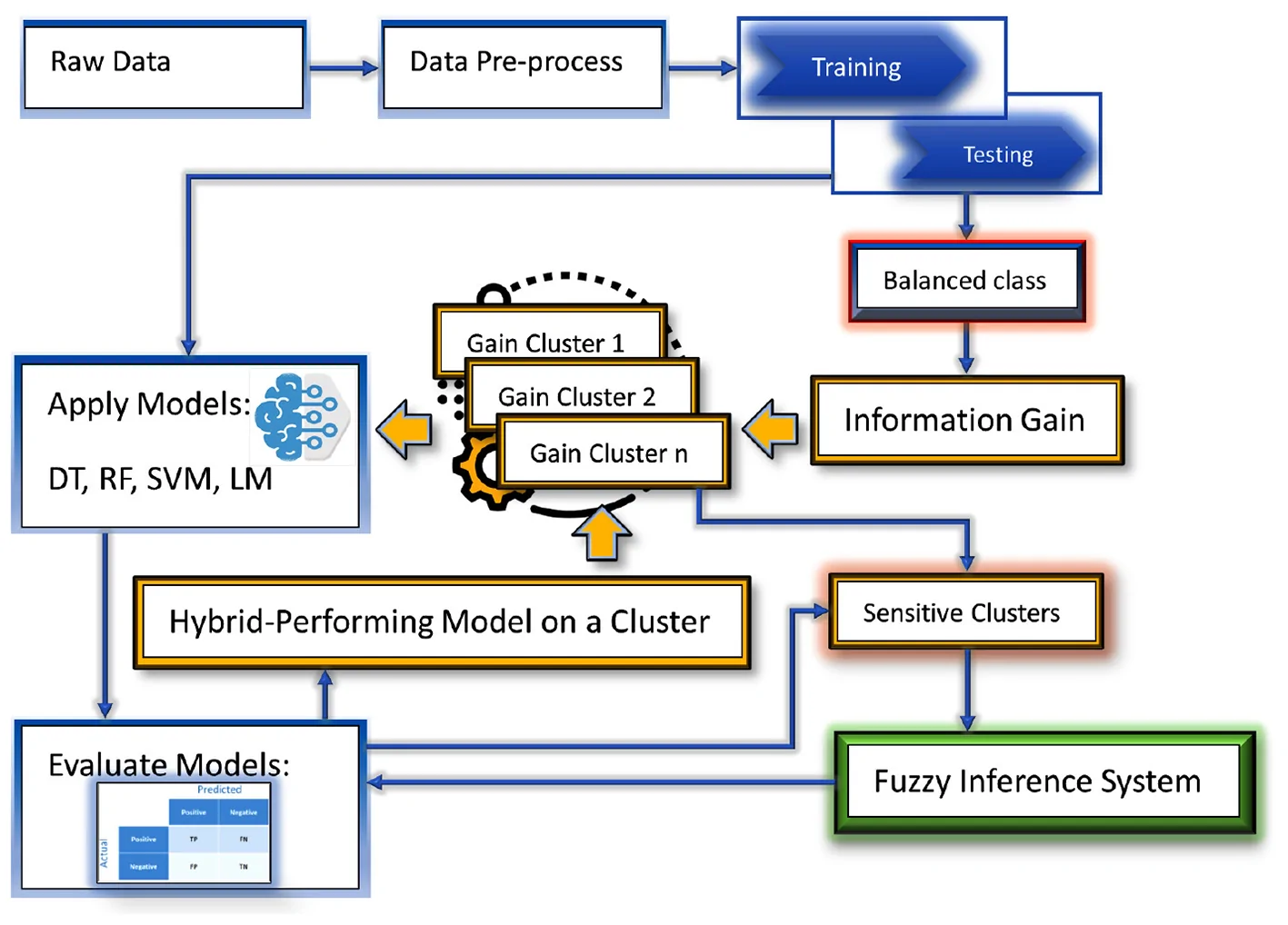
Intelligent learning Fuzzy Inference System framework.

Algorithm 1 Adaptive IFI–ML (entropy-guided hybrid ML + Sugeno FIS). 
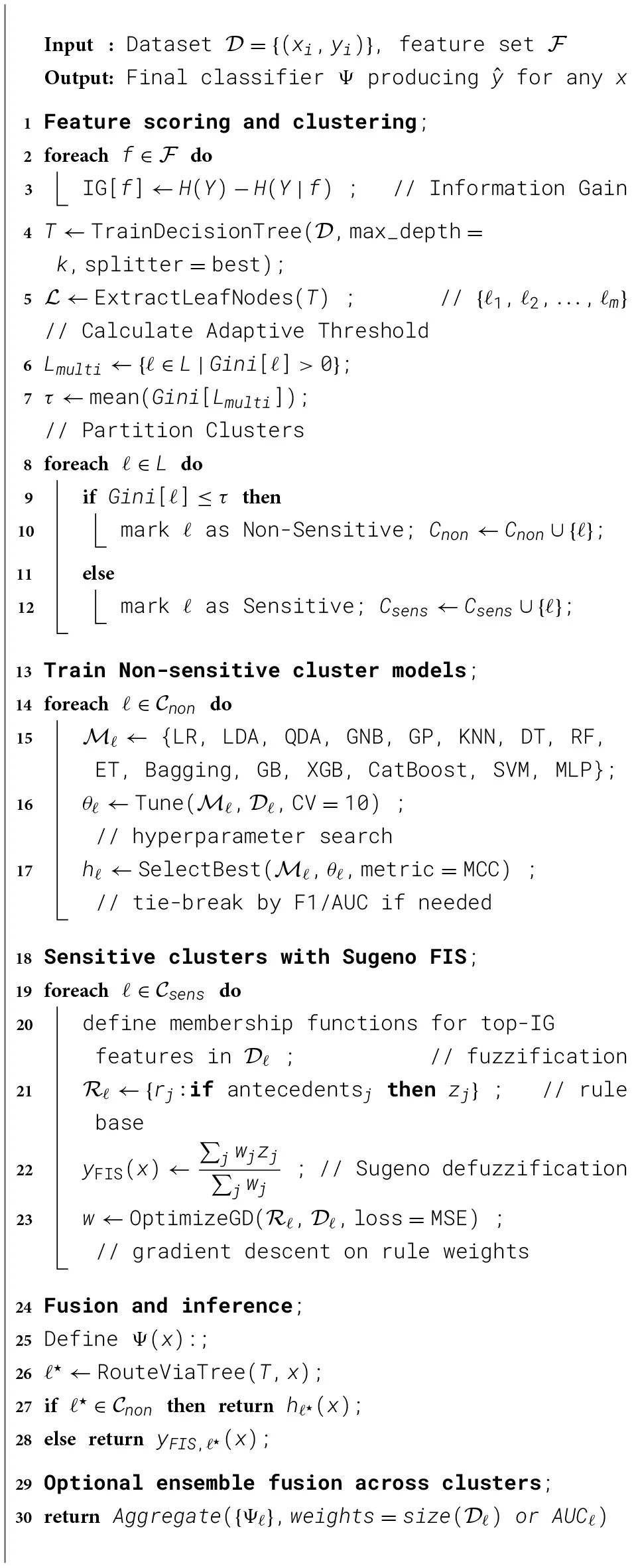

**Table 1:** Summary of UCI Parkinson's voice datasets.

Dataset	Reference	Healthy	Patients	Features	Instances	Class 0	Class 1
*D*_1_	Little, 2007	8	23	23	195	48	147
*D*_1_	Sakar et al., 2018	64	188	754	756	192	564

*D*_1_ is a smaller PD voice dataset with 23 acoustic features; *D*_2_ is a larger heterogeneous dataset including Tunable Q-factor Wavelet Transform (TQWT) and Mel-Frequency Cepstral Coefficients (MFCC) descriptors. Dependent variable *Y* ∈ {0, 1} (where 1 represents PD patients and 0 controls).

Figure 1 shows the IFI-ML learning framework's high-level architecture and the main steps for optimizing sensitive PD clusters with critical boundary uncertainty. The first step in the input stage is to process raw data through data preprocessing. Then, in the balancing and grouping stage, the Parkinson's datasets are put into information gain-based clustering, which creates gain clusters (*C*_1_, *C*_2_, ..., *C*_*n*_) using high-IG features. There are two types of tree-based clusters: non-sensitive and sensitive clusters. The ML model [Logistic regression (LR), Linear discriminant analysis (LDA), Quadratic discriminant analysis (QDA), Gaussian naive bayes (GNB), K-nearest neighbors (KNN), Decision tree [DT], Support vector machine (SVM), Random forest (RF), Extra tree (ET), Bagging, Gradient boosting (GB), eXtreme gradient boosting (XGBoost), Categorical boosting (CatBoost), Gaussian process (GP), and Multi-layer perceptron (MLP)] is used on non-sensitive clusters to use the hybrid performance models. Then, Sugeno-FIS handles the sensitive clusters to ensure that the predictions are strong. The ML models do not handle the sensitive clusters well because they have high impurities. This adaptive data partitioning lets the framework focus on where the resources are needed most and reduces noisy features impacts on Parkinson's classification.

Finally, this combination of ML and FIS models combines predictions, and fuzzy optimization makes them stronger. Followed by Figure 2 shows the flowchart of IFI-ML end-to-end execution, starting with Parkinson data and branching based on how sensitive the clusters are. If Gini impurity is greater than τ, FIS will handle the respective sensitive cluster by tuning the inference rules. If not, the samples will be handled by ML classifiers that work on the partitioned clusters. These numbers together show that IFI-ML is a combination of different methods: ML for scalable classification, FIS for resolving sensitive uncertainty, and clustering for balancing out imbalances.

**Figure 2:**
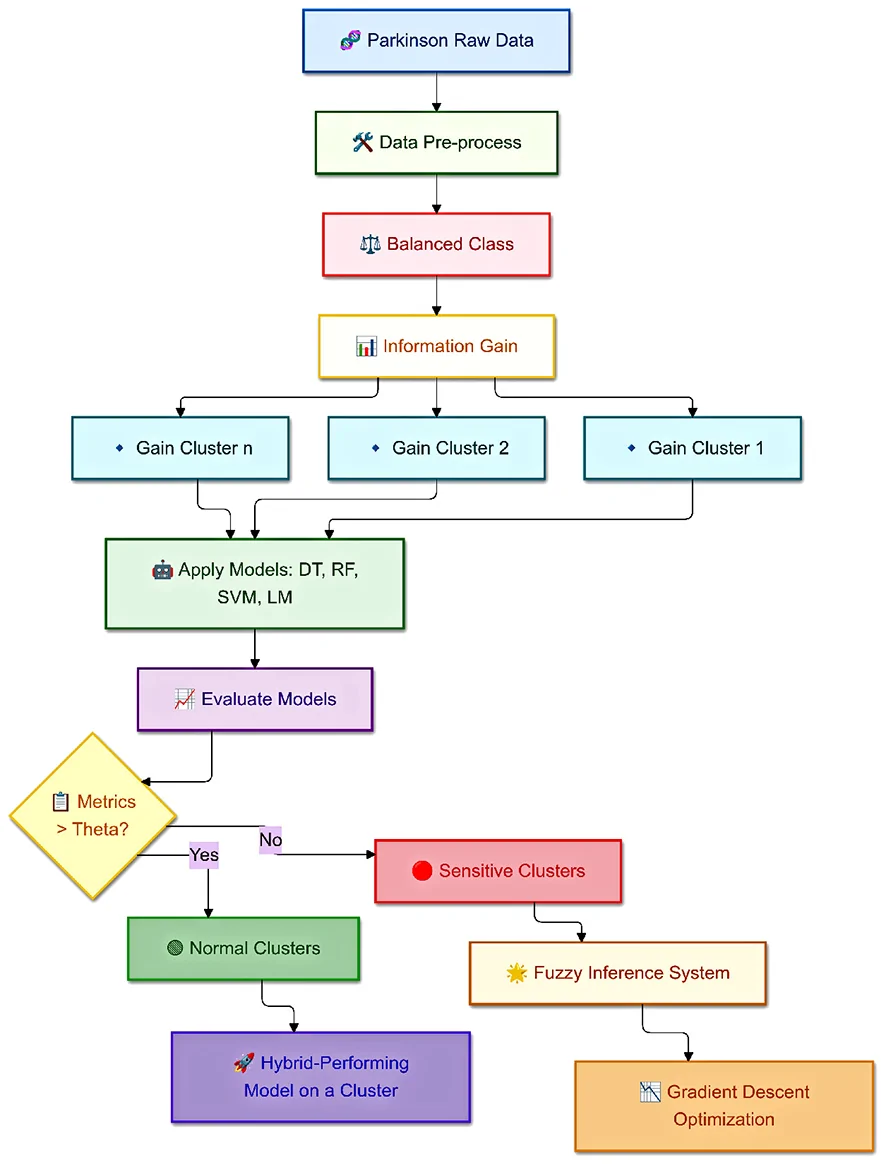
Flowchart of IFI-ML.

### 2.1 Balancing and grouping

PD samples are balanced by target class, grouped via high-information-gain features, and fused from the best-performing machine learning models. The entropy measure *H*(*Y*) uses the calculation as *H*(*Y*) = −∑*p*(*y*)log_2_*p*(*y*). The information gain *IG* for feature selection is measured as: *IG* = *H*(*Y*) − *H*(*Y*|*X*) where *H*(*Y*) is the entropy of the target variable *Y*, and *H*(*Y*|*X*) is the conditional entropy of *Y* given feature *X*. Here the dependent variable *Y* ∈ {0, 1} (where 1 represents PD patients and 0 represents healthy controls) and *X* is the independent variable. This approach addresses the issues of severe data leakage and highly biased, too-optimistic performance that arise when conventional workflows apply SMOTE or Random Over-Sampling (ROS) globally to the entire dataset before partition splitting. Any necessary class balancing is performed exclusively within the isolated training partition of each active fold. This prevents the generation of synthetic minority samples that overlap with, or leak details of, the held-out test fold's decision boundaries. By isolating localized boundary uncertainty and routing those specific samples directly to the Sugeno FIS pathway, IFI-ML completely avoids the class distribution distortions, local class overlaps, and boundary-blurring typical of global resampling methods.

### 2.2 Intelligent model construction

In this stage, refined PD samples are used to train and test various machine learning models. High-performing models are selected based on their classification accuracy. This intelligent model combines the best-performing machine learning models from each cluster, enhancing the overall performance. The intelligent model's aggregated output is computed as, $f\left(x\right)=\overset{n}{\underset{i=1}{\sum }}p_{i}h_{i}\left(x\right)$ where *h*_*i*_(*x*) represents the predictions of the *i*^*th*^ model, and *p*_*i*_ is the proportion of samples assigned to that model.

### 2.3 Optimization with Sugeno Fuzzy Inference System (FIS)

Sensitive clusters that are not well-trained by machine learning models are optimized using the Sugeno FIS. The FIS adjusts classification rules and feature importance, employing the gradient descent method for rule optimization. The Sugeno FIS prediction *y* is expressed as: $y=\frac{\overset{n}{\underset{i=1}{\sum }}w_{i}\cdot f_{i}}{\overset{n}{\underset{i=1}{\sum }}w_{i}}$ where *w*_*i*_ represents the weight, and *f*_*i*_ represents the output of the *i*^*th*^ rule. The gradient descent method updates the parameters as: $\theta_{t+1}=\theta_{t}-\eta \frac{\partial J}{\partial \theta_{t}}$, where η is the learning rate, θ_*t*_ denotes the parameters at iteration *t*, and *J* is the loss function.

### 2.4 Threshold selection (τ) and adaptive partitioning

To ensure the framework is data-adaptive rather than relying on a fixed heuristic, we propose a dynamic calculation for the Gini threshold τ. This threshold is defined as the mean Gini impurity of all multi-label leaf nodes in the partitioning tree, representing the baseline boundary uncertainty specific to the dataset:


$$\begin{array}{c} \tau =\frac{\sum_{i=1}^{N}Gini\left(\ell_{multi,i}\right)}{N} \end{array}$$ (1)

where *N* is the total number of multi-label leaf nodes (nodes where *Gini* > 0) and *Gini*(ℓ_*multi, i*_) denotes the impurity of the *i*-th multi-label node. This adaptive selection statistically supports the handling logic between Machine Learning (ML) and the Fuzzy Inference System (FIS) in the feature space's localized entropy. By calculating the Gini threshold dynamically using Equation 1, the boundary threshold adaptively adjusts to the specific local entropy of the training fold.

Moreover, to eliminate representation leakage, the Information Gain (*IG*) feature selection and Gini-tree partitioning tree (*T*) training are never performed globally on the entire dataset. Instead, they are executed dynamically inside each outer cross-validation fold using only the active training split data *D*. Because the outer test fold is completely withheld prior to these stages, the hierarchical structure of T and the selected feature subsets contain absolutely zero prior knowledge of the test fold's distribution or class labels, satisfying the most stringent conditions for avoiding data leakage.

### 2.5 Intelligent models and FIS

The fusion of intelligent models with the Sugeno FIS enhances the robustness of the classification system by effectively managing sensitive clusters and aggregating predictions to improve overall accuracy.

### 2.6 Model interpretation

Clinical decision-makers lack confidence in machine learning models due to their complexity. Shapley values from game theory are used in SHAP (Shapley Additive exPlanations) model interpretation. It calculates marginal contributions across all feature subsets and weighted averages them to quantify feature effects on predictions. Summary plots show global feature effects, both feature importance rankings (vertical axis) and sample SHAP values (horizontal axis). The color gradient, which maps feature magnitudes (red for high, blue for low), provides an intuitive view of feature-prediction relationships. Force plots show local interpretability by cumulatively shifting the model's output from a baseline value (typically the average prediction) to the final prediction for individual samples by each feature's SHAP value (red/blue arrows proportional to contribution magnitude). This shows how specific features affect instance classification decisions.

## 3 Results

We evaluated fifteen machine learning (*ML*) algorithms combined with the proposed Intelligent Fuzzy Inference-Machine Learning framework (*IFI* − *ML*) for Parkinson's disease (*PD*) detection on two voice-based datasets, *D*_1_ and *D*_2_; its description is shown in Table 1. The results of 10-fold cross-validation are presented in Tables 2, 3 presents the performance comparison of all models with the Synthetic Minority Over-sampling Technique (*SMOTE*) and Random Over-sampling (*ROS*) class-balancing techniques. The accuracy and specificity metrics achieved by *IFI* − *ML* are compared with baseline and balancing results in the bar chart of Figure 3 and the radar chart of Figure 4 for Datasets *D*_1_ and *D*_2_, respectively. We report the performance metrics of all classifiers with the *IFI* − *ML* framework shown in Table 4. The single-label partitions, multi-label non-sensitive partitions, and multi-label sensitive partitions are visualized clearly in Figures 5, 6, and then their respective inferential statistics are displayed in Table 5. Figures 7, 8 illustrate the mean absolute SHAP values for these partitions across both datasets. All computational pipelines were executed in Python using Scikit-learn, with visualizations generated via Matplotlib and Seaborn on a Windows 11 system equipped with a 12^th^ Gen Intel^®^ Core™ i5 1235U and 16 GB of RAM.

**Table 2:** Performance of ML models on the Parkinson's disease datasets D_1_ & D_2_ using 10 cross-validation.

IFI-ML	*LR*	*LDA*	*QDA*	*GNB*	*KNN*	*DT*	*SVM*	*RF*	*ET*	*Bagging*	*GB*	*XGBoost*	*CatBoost*	*GP*	*MLP*
PD − D_1_ Dataset (10 CV)															
Accuracy	84.42%	81.29%	83.92%	66.45%	81.42%	79.26%	84.45%	83.37%	88.03%	83.92%	88.05%	89.16%	82.87%	80.26%	90.16%
Precision	85.89%	87.76%	86.71%	91.84%	84.05%	87.50%	84.62%	87.34%	89.17%	87.66%	89.61%	90.38%	85.19%	85.16%	93.79%
TPR	95.24%	87.76%	93.20%	61.22%	93.20%	85.71%	97.28%	93.88%	95.24%	91.84%	93.88%	95.92%	93.88%	89.80%	92.52%
TNR	52.08%	62.50%	56.25%	83.33%	45.83%	62.50%	45.83%	58.33%	64.58%	60.42%	66.67%	68.75%	50%	52.08%	81.25%
FPR	47.92%	37.50%	43.75%	16.67%	54.17%	37.50%	54.17%	41.67%	35.42%	39.58%	33.33%	31.25%	50%	47.92%	18.75%
FNR	4.96%	12.24%	6.80%	38.78%	6.80%	14.29%	2.72%	6.12%	4.76%	8.16%	6.12%	4.08%	6.12%	10.20%	7.48%
F1 Score	90.32%	87.76%	89.84%	73.47%	88.39%	86.60%	90.51%	90.49%	92.11%	89.70%	91.69%	93.07%	89.32%	87.42%	93.15%
MCC	0.5504	0.5026	0.5432	0.3839	0.4540	0.4726	0.5463	0.5736	0.6506	0.5524	0.64	0.6964	0.5041	0.4468	0.7277
AUC-ROC	0.8492	0.805	0.8379	0.8114	0.889	0.7273	0.7565	0.8788	0.9163	0.8711	0.905	0.9289	0.8882	0.8313	0.9615
AUC-PR	0.9488	0.9129	0.9411	0.9281	0.9646	0.9156	0.8907	0.9571	0.9693	0.9496	0.9608	0.9736	0.9611	0.9402	0.9867
PD − D_2_ Dataset (10 CV)															
Accuracy	80.57%	81.75%	72.24%	75.66%	81.74%	78.04%	83.99%	84.52%	82.26%	84.51%	84.52%	84.79%	85.45%	80.81%	82.68%
Precision	86.64%	86.60%	79.30%	86.40%	83.60%	82.75%	83.61%	83.72%	84.46%	85.29%	85.92%	85.92%	86.61%	82.89%	86.68%
TPR	87.41%	89.36%	84.93%	79.96%	93.97%	88.48%	97.70%	95.74%	95.39%	91.49%	95.21%	95.21%	95.21%	93.62%	91.13%
TNR	60.42%	59.38%	34.90%	63.02%	45.83%	45.83%	43.75%	45.31%	48.44%	53.65%	54.17%	54.17%	56.77%	43.23%	58.85%
FPR	39.58%	40.62%	65.10%	36.98%	54.17%	54.17%	56.25%	54.69%	51.56%	46.35%	45.83%	45.83%	43.23%	56.67%	41.15%
FNR	12.59%	10.64%	15.07%	20.04%	6.03%	11.52%	2.30%	4.26%	4.61%	8.51%	4.79%	4.79%	4.79%	6.38%	8.87%
F1 Score	87.03%	87.96	82.02	83.06	88.48	85.52	90.11	89.33	89.59	88.28	90.33	90.33	90.71	87.93	88.85
MCC	0.8055	0.8236	0.6842	0.7419	0.7885	0.6963	0.8192	0.8477	0.8476	0.8195	0.8676	0.8720	0.8821	0.7218	0.8266
AUC-ROC	0.9150	0.9240	0.8391	0.8588	0.8791	0.8294	0.9172	0.9381	0.9406	0.9083	0.9424	0.9417	0.9546	0.8701	0.9276
AUC-PR	0.4825	0.5040	0.2153	0.4047	0.4710	0.3717	0.5394	0.5049	0.5238	0.4914	0.5679	0.5679	0.5891	0.4404	0.5291

**Table 3:** Performance of overall results on the PD D_1_ & D_2_ (10 CV) with SMOTE and ROS balancing.

IFI-ML	*LR*	*LDA*	*QDA*	*GNB*	*KNN*	*DT*	*SVM*	*RF*	*ET*	*Bagging*	*GB*	*XGBoost*	*CatBoost*	*GP*	*MLP*
PD − D_1_ SMOTE															
Accuracy	76.13%	74.71%	84.47%	68.53%	83%	83.95%	77.68%	83.92%	88.55%	79.24%	85.53%	87.61%	85.11%	79.76%	90.68%
Precision	87.97%	90.83%	85.45%	92.16%	94.53%	89.86%	88.24%	88.51%	92.57%	89.36%	89.40%	91.84%	90.41%	90.30%	95%
TPR	79.59%	74.15%	95.92%	63.95%	82.31%	90.48%	81.63%	89.12%	93.20%	85.71%	91.84%	91.84%	89.80%	82.31%	90.48%
TNR	66.67%	77.08%	50%	83.33%	85.42%	68.75%	66.67%	64.58%	77.08%	68.75%	66.67%	75%	70.83%	72.92%	85.42%
FPR	33.33%	22.92%	50%	16.67%	14.58%	31.25%	33.33%	35.42%	22.92%	31.25%	33.33%	25%	29.17%	27.08%	14.58%
FNR	20.41%	25.85%	4.08%	36.05%	17.69%	9.52%	18.37%	10.88%	6.80%	14.29%	8.16%	8.16%	10.20%	17.69%	9.52%
F1 Score	83.57%	81.65%	90.38%	75.50%	88%	90.17%	84.81%	88.81%	92.88%	87.50%	90.60%	91.84%	90.10%	86.12%	92.68%
MCC	0.4279	0.4536	0.5482	0.4078	0.6143	0.5965	0.4529	0.5408	0.7078	0.5243	0.6029	0.6684	0.6021	0.5131	0.7265
AUC-ROC	0.8279	0.8571	0.8234	0.8090	0.8831	0.7670	0.8203	0.8945	0.9205	0.8554	0.9055	0.9269	0.9131	0.8588	0.9569
AUC-PR	0.9378	0.9523	0.9402	0.9273	0.9608	0.9291	0.9355	0.9611	0.9722	0.9432	0.9607	0.9740	0.9691	0.9492	0.9855
PD − D_1_ Random over-sampler															
Accuracy	76.08%	76.16%	84.45%	67.47%	81.42%	83.55%	77.74%	82.89%	88.55%	81.84%	85.55%	89.13%	86.08%	74.08%	90.71%
Precision	86.86%	88.55%	85.89%	92%	93.70%	87.58%	86.62%	87.10%	88.82%	88%	87.34%	90.91%	87.97%	85.40%	93.01%
TPR	80.95%	78.91%	95.24%	62.59%	80.95%	91.16%	83.67%	91.84%	97.28%	89.80%	93.88%	95.24%	94.56%	79.59%	90.48%
TNR	62.50%	68.75%	52.08%	83.33%	83.33	60.42%	60.42%	58.33%	62.50%	62.50%	58.33%	70.83%	60.42%	58.33%	79.17%
FPR	37.50%	31.25%	47.92%	16.67%	16.67%	39.58%	39.58%	41.67%	37.50%	37.50%	41.67%	29.17%	39.58%	41.67%	20.83%
FNR	19.05%	21.09%	4.76%	37.41%	19.05%	8.84%	16.33%	8.16%	2.72%	10.20%	6.12%	4.76%	5.44%	20.41%	9.52%
F1 Score	83.80%	83.45%	90.32%	74.49%	86.86%	89.33%	85.12%	89.40%	92.86%	88.89%	90.49%	93.02%	91.15%	82.39%	91.72%
MCC	0.4095	0.4372	0.5504	0.3957	0.5811	0.5404	0.4269	0.5352	0.6787	0.5347	0.5736	0.6985	0.6040	0.3574	0.6784
AUC-ROC	0.8141	0.8155	0.8228	0.8133	0.8815	0.7460	0.8493	0.8872	0.9179	0.8834	0.8825	0.9247	0.9209	0.8162	0.9576
AUC-PR	0.9323	0.9229	0.9372	0.9278	0.9593	0.9061	0.9474	0.9591	0.9706	0.9531	0.9494	0.9715	0.9726	0.9336	0.9857
PD − D_2_ SMOTE															
Accuracy	78.32%	77.65%	80.56%	72.35%	69.02%	76.71%	81.88%	82.53%	83.46%	82.92%	82.93%	85.04%	81.74%	78.56%	81.36%
Precision	86.50%	87.76%	81.87%	86.60%	91.04%	85.90%	84.94%	86.84%	86.04%	88.30%	88.40%	88.16%	87.90%	87.36%	87.06%
TPR	84.04%	81.38%	94.50%	74.47%	64.89%	82.09%	92.02%	90.07%	92.91%	88.30%	89.18%	92.38%	87.59%	83.33%	88.30%
TNR	61.46%	66.67%	38.54%	66.15%	81.25%	60.42%	52.08%	59.90%	55.73%	65.62%	65.62%	63.54%	64.58%	64.58%	61.46%
FPR	38.54%	33.33%	61.46%	33.85%	18.75%	39.58%	47.92%	40.10%	44.27%	34.38%	34.38%	36.46%	35.42%	35.42%	38.54%
FNR	15.96%	18.62%	5.50%	25.53%	35.11%	17.91%	7.98%	9.93%	7.09%	11.70%	10.82%	7.62%	12.41%	16.67%	11.70%
F1 Score	85.25%	84.45%	87.74%	80.08%	75.78%	83.95%	88.34%	88.42%	89.34%	88.30%	88.79%	90.22%	87.74%	85.30%	87.68%
MCC	0.4435	0.4530	0.4159	0.3686	0.4025	0.4090	0.4876	0.5199	0.5349	0.5392	0.5529	0.5893	0.5199	0.4604	0.5047
AUC-ROC	0.8031	0.8213	0.6986	0.7523	0.7950	0.6935	0.8235	0.8673	0.8592	0.8679	0.8794	0.8854	0.8685	0.8222	0.8402
AUC-PR	0.9140	0.9262	0.8324	0.8650	0.8957	0.8293	0.9277	0.9499	0.9458	0.9410	0.9521	0.9524	0.9490	0.9284	0.9332
PD − D_2_ Random over-sampler															
Accuracy	78.06%	79.11%	79.89%	75.27%	77.91%	74.21%	82.54%	82.80%	82.93%	82.53%	85.44%	84.25%	80.41%	79.61%	81.36%
Precision	86.58%	87.45%	82.55%	86.18%	85.38%	86.10%	85.76%	85.74%	83.56%	84.83%	87.36%	87.15%	86.49%	85.42%	87.23%
TPR	83.51%	84.04%	93.09%	79.61%	84.93%	79.08%	91.84%	92.73%	96.45%	90.25%	94.33%	92.55%	87.41%	88.30%	87.23%
TNR	61.98%	64.58%	42.19%	62.50%	57.29%	62.50%	55.21%	54.69%	44.27%	52.60%	59.90%	59.90%	59.90%	55.73%	62.50%
FPR	38.02%	35.42%	57.81%	37.50%	42.71%	37.50%	44.79%	45.31%	55.73%	47.40%	40.10%	40.10%	40.10%	44.27%	37.50%
FNR	16.49%	15.96%	6.91%	20.39%	15.07%	20.92%	8.16%	7.27%	3.55%	9.75%	5.67%	7.45%	12.59%	11.70%	12.77%
F1 Score	85.02%	85.71%	87.50%	82.76%	85.16%	82.44%	88.70%	89.10%	89.55%	87.46%	90.71%	89.77%	86.95%	86.84%	87.23%
MCC	0.4408	0.4698	0.4202	0.3960	0.4201	0.3897	0.5110	0.5229	0.5126	0.4609	0.5963	0.5628	0.4781	0.4562	0.4973
AUC-ROC	0.8004	0.8201	0.7050	0.7420	0.7731	0.7554	0.8159	0.8438	0.8500	0.8431	0.8747	0.8788	0.8476	0.7711	0.8276
AUC-PR	0.9106	0.9247	0.8360	0.8582	0.8759	0.8746	0.9187	0.9371	0.9423	0.9290	0.9461	0.9511	0.9404	0.8925	0.9234

**Figure 3:**
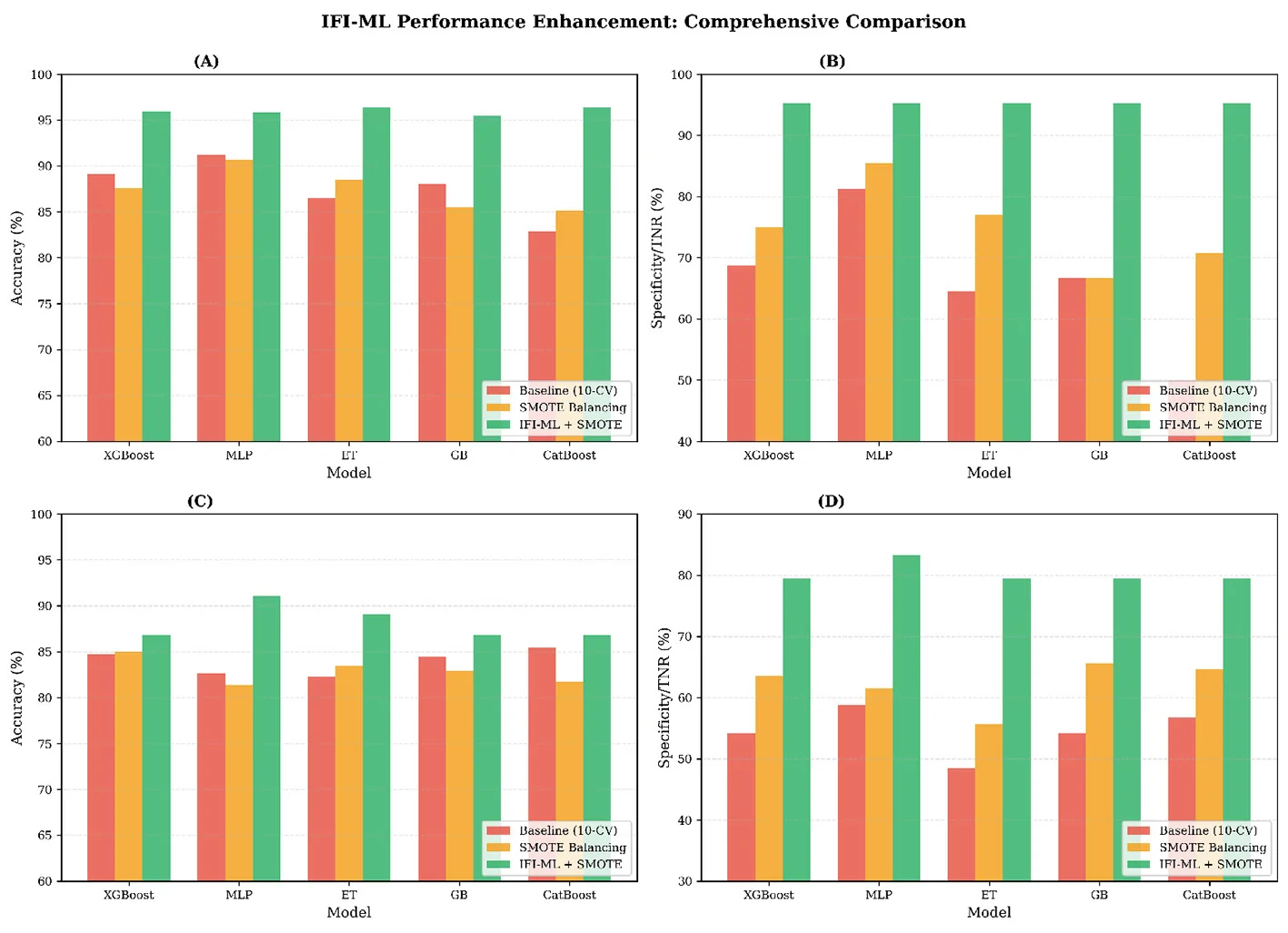
IFI-ML performance enhancement of accuracy and specificity. **(A)** Dataset 1: Accuracy progression. **(B)** Dataset 1: Specificity enhancement. **(C)** Dataset 2: Accuracy progression. **(D)** Dataset 2: Specificity enhancement.

**Figure 4:**
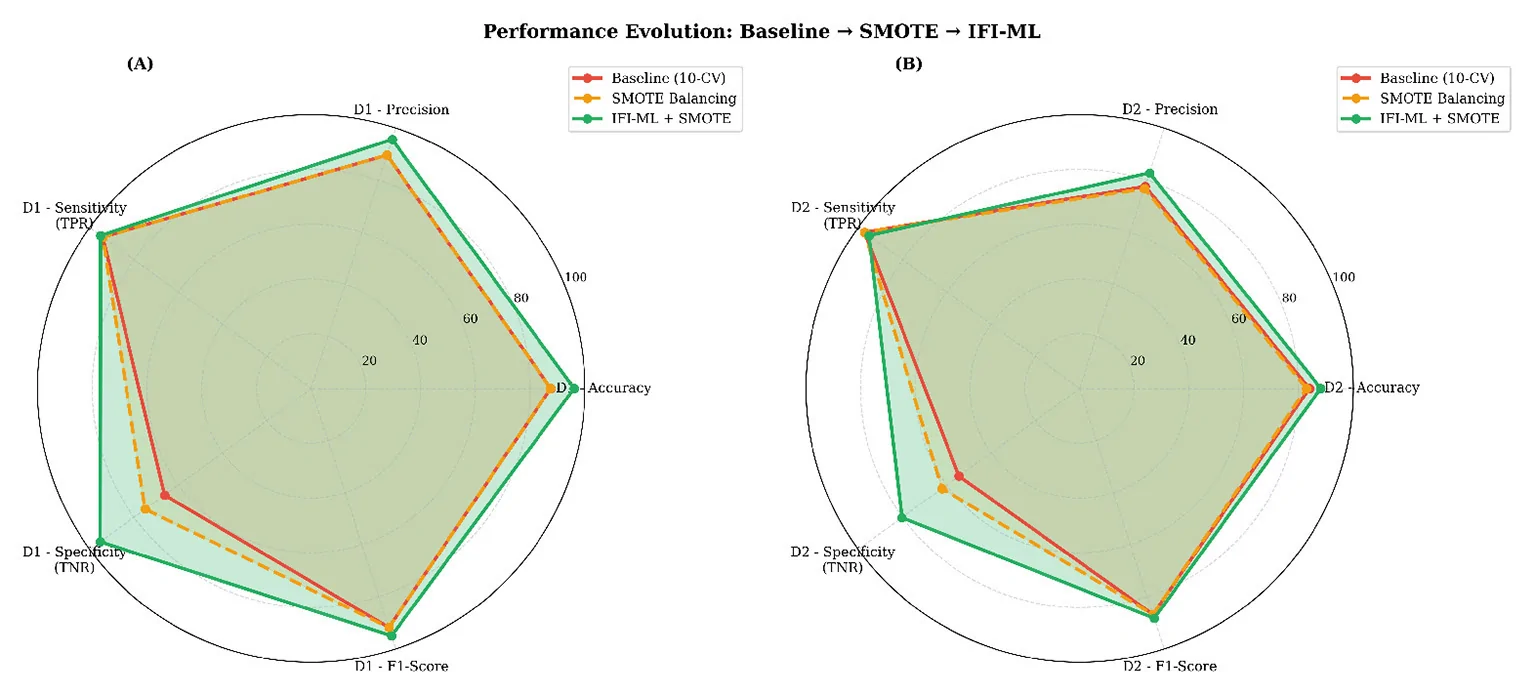
Performance evolution. **(A)** Dataset 1: Multi-metric performance (average of top five models). **(B)** Dataset 2: Multi-metric performance (average of top five models).

**Table 4:** Performance of IFI-ML − overall results on the PD D_1_ & D_2_ with 10 CV and balancing.

IFI-ML	*LR*	*LDA*	*QDA*	*GNB*	*KNN*	*DT*	*SVM*	*RF*	*ET*	*Bagging*	*GB*	*XGBoost*	*CatBoost*	*GP*	*MLP*
Accuracy	95.88%	96.43%	95.34%	95.34%	95.88%	94.95%	96.43%	96.43%	96.43%	95.49%	95.49%	95.96%	96.43%	96.35%	95.88%
Precision	95.65%	95.65%	95.16%	95.16%	95.16%	95.65%	95.65%	95.98%	95.65%	95.65%	95.65%	95.65%	95.65%	95.16%	95.65%
TPR	94.80%	95.90%	94.80%	94.80%	95.90%	92.89%	95.90%	95.90%	95.90%	93.98%	93.98%	94.80%	95.90%	96.72%	94.80%
TNR	95.29%	95.29%	94.23%	94.23%	94.23%	95.29%	95.29%	95.29%	95.29%	95.29%	95.29%	95.29%	95.29%	94.23%	95.29%
FPR	4.71%	4.71%	5.77%	5.77%	5.77%	4.71%	4.71%	4.71%	4.71%	4.71%	4.71%	4.71%	4.71%	5.77%	4.71%
FNR	5.27%	4.28%	5.27%	5.27%	4.28%	7.26%	4.28%	4.28%	4.28%	6.27%	6.27%	5.27%	4.28%	3.28%	5.27%
F1 Score	94.84%	95.49%	94.49%	94.49%	95.14%	93.71%	95.49%	95.61%	95.49%	94.37%	94.37%	94.84%	95.49%	95.61%	94.84%
MCC	0.9168	0.9264	0.9082	0.9082	0.9179	0.8997	0.9264	0.9275	0.9264	0.9093	0.9093	0.9175	0.9264	0.9261	0.9168
AUC-ROC	0.9772	0.9526	0.9763	0.9704	0.9658	0.948	0.9772	0.9822	0.9836	0.9781	0.9726	0.9754	0.9809	0.9809	0.9799
AUC-PR	0.9605	0.9404	0.9605	0.9495	0.944	0.9232	0.9605	0.9633	0.9649	0.9583	0.9548	0.9597	0.9633	0.9633	0.9622
PD − D_1_ IFI-ML− Random over-sampler															
Accuracy	95.88%	96.43%	95.34%	95.34%	96.35%	95.49%	96.43%	95.88%	95.41%	96.98%	95.49%	95.96%	96.43%	96.35%	94.87%
Precision	95.65%	95.65%	95.16%	95.16%	95.16%	96.47%	95.65%	95.16%	95.16%	96.47%	95.65%	95.65%	95.65%	95.16%	95.16%
TPR	94.80%	95.90%	94.80%	94.80%	96.72%	92.89%	95.90%	95.90%	95.08%	95.90%	93.98%	94.80%	95.90%	96.72%	93.98%
TNR	95.29%	95.29%	94.23%	94.23%	94.23%	96.34%	95.29%	94.23%	94.23%	96.34%	95.29%	95.29%	95.29%	94.23%	94.23%
FPR	4.71%	4.71%	5.77%	5.77%	5.77%	3.66%	4.71%	5.77%	5.77%	3.66%	4.71%	4.71%	4.71%	5.77%	5.77%
FNR	5.27%	4.28%	5.27%	5.27%	3.28%	7.26%	4.28%	4.28%	5.27%	4.28%	6.27%	5.27%	4.28%	3.28%	6.27%
F1 Score	94.84%	95.49%	94.49%	94.49%	95.61%	94.18%	95.49%	95.14%	94.67%	95.96%	94.37%	94.84%	95.49%	95.61%	94.02%
MCC	0.9168	0.9264	0.9082	0.9082	0.9261	0.9093	0.9264	0.9179	0.9096	0.936	0.9093	0.9175	0.9264	0.9261	0.9
AUC-ROC	0.9772	0.9526	0.9763	0.9704	0.9713	0.9535	0.9772	0.9809	0.9836	0.9726	0.9576	0.9754	0.9809	0.9809	0.9799
AUC-PR	0.9605	0.9404	0.9605	0.9495	0.9489	0.9314	0.9605	0.9633	0.9649	0.9548	0.9368	0.9597	0.9633	0.9633	0.9622
PD − D_2_ IFI-ML− SMOTE															
Accuracy	88.52%	86.50%	91.26%	84.47%	87.81%	86.60%	90.95%	89.03%	89.08%	86.85%	86.80%	86.85%	86.85%	88.98%	91.06%
Precision	94.95%	94.68%	93.23%	95.09%	95.40%	94.59%	93.79%	94.04%	94.23%	94.60%	94.59%	94.60%	94.60%	94.95%	95.23%
TPR	91.25%	89.02%	95.79%	85.63%	88.76%	89.53%	95.34%	92.47%	93.30%	89.72%	89.66%	89.72%	89.72%	91.64%	93.74%
TNR	81.72%	80.48%	75.83%	83.71%	87.96%	79.49%	77.79%	79.00%	79.51%	79.49%	79.49%	79.49%	79.49%	82.47%	83.22%
FPR	18.28%	19.52%	24.17%	16.29%	12.04%	20.51%	22.21%	20.50%	20.49%	20.51%	20.51%	20.51%	20.51%	17.53%	16.53%
FNR	8.75%	10.98%	4.21%	14.37%	11.24%	10.73%	4.66%	7.22%	7.15%	10.28%	10.34%	10.28%	10.28%	8.36%	5.43%
F1 Score	91.28%	89.12%	94.13%	86.12%	90.78%	89.24%	94.03%	92.57%	92.10%	89.48%	89.44%	89.48%	89.48%	91.85%	93.89%
PD − D_1_ IFI-ML− SMOTE															
MCC	0.752	0.7035	0.7253	0.7102	0.7573	0.6873	0.7451	0.7372	0.7367	0.6944	0.6897	0.6944	0.6944	0.7528	0.7792
AUC-ROC	0.9471	0.907	0.8653	0.8603	0.8976	0.8448	0.9067	0.909	0.9085	0.8566	0.8777	0.9146	0.8674	0.9015	0.9291
AUC-PR	0.9703	0.9523	0.9212	0.9297	0.9462	0.9242	0.9529	0.9511	0.955	0.9286	0.9367	0.9582	0.9425	0.9535	0.964
PD − D_2_ IFI-ML− Random over-sampler															
Accuracy	88.68%	86.65%	92.73%	84.47%	91.87%	86.55%	90.90%	89.49%	90.09%	86.60%	86.55%	86.85%	86.85%	92.17%	91.31%
Precision	94.95%	94.66%	93.70%	95.28%	94.39%	94.59%	93.93%	93.81%	93.91%	94.25%	93.62%	94.60%	94.60%	95.12%	94.85%
TPR	91.44%	89.21%	97.19%	85.38%	95.40%	89.27%	95.15%	93.36%	95.02%	89.72%	89.59%	89.72%	89.72%	96.42%	94.25%
TNR	81.72%	80.48%	76.80%	84.71%	82.03%	76.55%	78.28%	78.03%	77.04%	79.49%	78.26%	79.49%	79.49%	84.46%	82.98%
FPR	18.28%	19.52%	23.20%	15.29%	17.97%	20.51%	21.72%	21.73%	22.46%	21.98%	21.98%	20.51%	20.51%	20.43%	17.52%
FNR	8.56%	10.79%	2.81%	14.62%	4.60%	10.73%	4.85%	7.28%	3.83%	10.41%	10.41%	10.28%	10.28%	7.98%	5.24%
F1 Score	91.46%	89.39%	95.08%	85.88%	94.65%	88.95%	93.96%	91.70%	93.81%	89.19%	89.30%	89.48%	89.48%	94.95%	93.74%
MCC	0.7533	0.7022	0.7556	0.7189	0.7796	0.6647	0.7471	0.7005	0.7314	0.6842	0.676	0.6944	0.6944	0.7704	0.7767
AUC-ROC	0.9466	0.9079	0.9035	0.8655	0.903	0.8306	0.9134	0.9081	0.9214	0.8463	0.8605	0.9051	0.8555	0.8745	0.935
AUC-PR	0.9701	0.9524	0.9389	0.9316	0.9471	0.9235	0.9556	0.9552	0.9567	0.9317	0.9333	0.9559	0.9364	0.944	0.965

**Figure 5:**
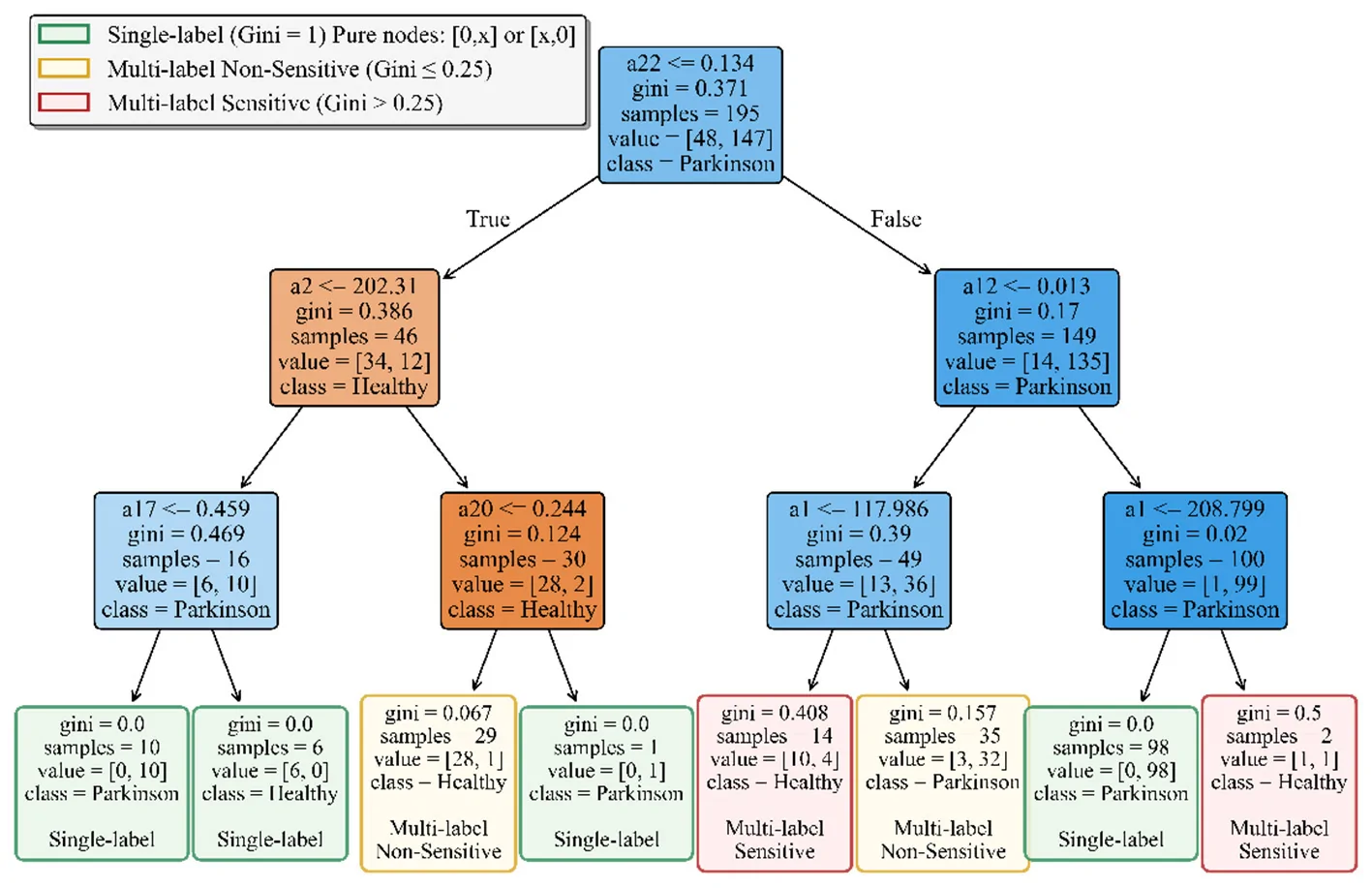
Parkinson's disease—D_1_ Gini impurity visualization.

**Figure 6:**
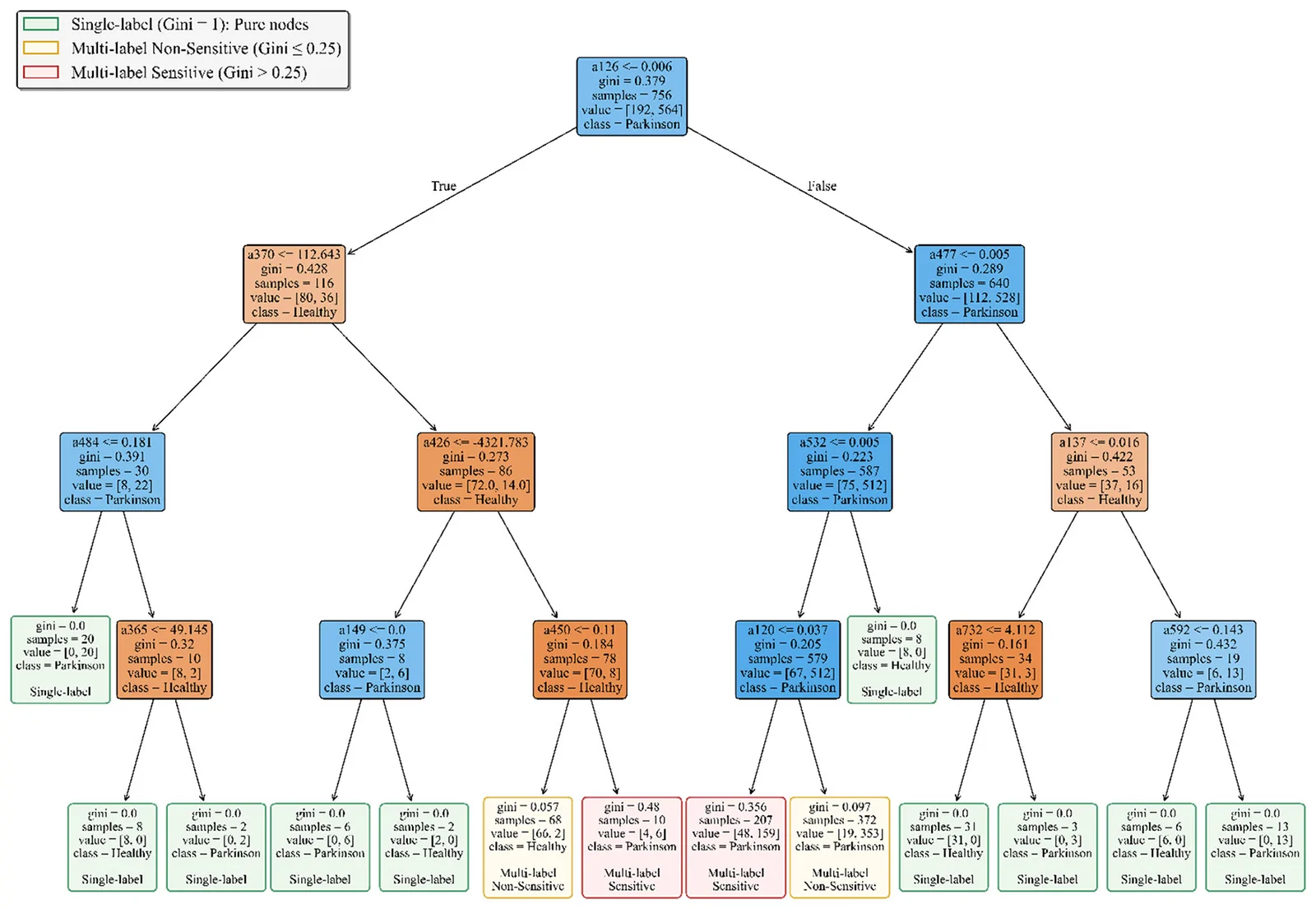
Parkinson's disease - D_2_ Gini impurity visualization.

**Table 5:** Leaf node summary and IFI-ML for Parkinson's disease D_1_ and D_2_.

Leaf node	Healthy	Parkinson	Total	Gini	Leaf type	IFI-ML	Rule path
Parkinson's disease D_1_							
3	0	10	10	0.000	Single-label	DT	PPE (*a*_22_) ≤ 0.134, MDVP:Fhi(Hz) (*a*_2_) ≤ 202.310, RPDE (*a*_17_) ≤ 0.459
4	6	0	6	0.000	Single-label	DT	PPE (*a*_22_) ≤ 0.134, MDVP:Fhi(Hz) (*a*_2_) ≤ 202.310, RPDE (*a*_17_) > 0.459
6	28	1	29	0.067	Multi-label Non-Sensitive	ML	PPE (*a*_22_) ≤ 0.134, MDVP:Fhi(Hz) (*a*_2_) > 202.310, spread2 (*a*_20_) ≤ 0.244
7	0	1	1	0.000	Single-label	DT	PPE (*a*_22_) ≤ 0.134, MDVP:Fhi(Hz) (*a*_2_) > 202.310, spread2 (*a*_20_) > 0.244
10	10	4	14	0.408	Multi-label Sensitive	FIS	PPE (*a*_22_) > 0.134, Shimmer:APQ5 (*a*_12_) ≤ 0.013, MDVP:Fo(Hz) (*a*_1_) ≤ 117.986
11	3	32	35	0.157	Multi-label Non-Sensitive	ML	PPE (*a*_22_) > 0.134, Shimmer:APQ5 (*a*_12_) ≤ 0.013, MDVP:Fo(Hz) (*a*_1_) > 117.986
13	0	98	98	0.000	Single-label	DT	PPE (*a*_22_) > 0.134, Shimmer:APQ5 (*a*_12_) > 0.013, MDVP:Fo(Hz) (*a*_1_) ≤ 208.799
14	1	1	2	0.500	Multi-label Sensitive	FIS	PPE (*a*_22_) > 0.134, Shimmer:APQ5 (*a*_12_) > 0.013, MDVP:Fo(Hz) (*a*_1_) > 208.799
Parkinson's disease D_2_							
3	0	20	20	0.000	Single-label	DT	std_delta_delta_log_energy (*a*_126_) ≤ 0.006, tqwt_entropy_shannon_dec_13 (*a*_370_) ≤ 112.643, tqwt_TKEO_std_dec_19 (*a*_484_) ≤ 0.181
5	8	0	8	0.000	Single-label	DT	std_delta_delta_log_energy (*a*_126_) ≤ 0.006, tqwt_entropy_shannon_dec_13 (*a*_370_) ≤ 112.643, tqwt_TKEO_std_dec_19 (*a*_484_) > 0.181, tqwt_entropy_shannon_dec_8 (*a*_365_) ≤ 49.145
6	0	2	2	0.000	Single-label	DT	std_delta_delta_log_energy (*a*_126_) ≤ 0.006, tqwt_entropy_shannon_dec_13 (*a*_370_) ≤ 112.643, tqwt_TKEO_std_dec_19 (*a*_484_) > 0.181, tqwt_entropy_shannon_dec_8 (*a*_365_) > 49.145
9	0	6	6	0.000	Single-label	DT	std_delta_delta_log_energy (*a*_126_) ≤ 0.006, tqwt_entropy_shannon_dec_13 (*a*_370_) > 112.643, tqwt_entropy_log_dec_33 (*a*_426_) ≤ -4321.783, Ed_9_coef (*a*_149_) ≤ 0.000
10	2	0	2	0.000	Single-label	DT	std_delta_delta_log_energy (*a*_126_) ≤ 0.006, tqwt_entropy_shannon_dec_13 (*a*_370_) > 112.643, tqwt_entropy_log_dec_33 (*a*_426_) ≤ -4321.783, Ed_9_coef (*a*_149_) > 0.000
12	66	2	68	0.057	Multi-label Non-Sensitive	ML	std_delta_delta_log_energy (*a*_126_) ≤ 0.006, tqwt_entropy_shannon_dec_13 (*a*_370_) > 112.643, tqwt_entropy_log_dec_33 (*a*_426_) > -4321.783, tqwt_TKEO_mean_dec_21 (*a*_450_) ≤ 0.110
13	4	6	10	0.480	Multi-label Sensitive	FIS	std_delta_delta_log_energy (*a*_126_) ≤ 0.006, tqwt_entropy_shannon_dec_13 (*a*_370_) > 112.643, tqwt_entropy_log_dec_33 (*a*_426_) > -4321.783, tqwt_TKEO_mean_dec_21 (*a*_450_) > 0.110
17	48	159	207	0.356	Multi-label Sensitive	FIS	std_delta_delta_log_energy (*a*_126_) > 0.006, tqwt_TKEO_std_dec_12 (*a*_477_) ≤ 0.005, tqwt_medianValue_dec_31 (*a*_532_) ≤ 0.005, std_7th_delta (*a*_120_) ≤ 0.037
18	19	353	372	0.097	Multi-label Non-Sensitive	ML	std_delta_delta_log_energy (*a*_126_) > 0.006, tqwt_TKEO_std_dec_12 (*a*_477_) ≤ 0.005, tqwt_medianValue_dec_31 (*a*_532_) ≤ 0.005, std_7th_delta (*a*_120_) > 0.037
19	8	0	8	0.000	Single-label	DT	std_delta_delta_log_energy (*a*_126_) > 0.006, tqwt_TKEO_std_dec_12 (*a*_477_) ≤ 0.005, tqwt_medianValue_dec_31 (*a*_532_) > 0.005
22	31	0	31	0.000	Single-label	DT	std_delta_delta_log_energy (*a*_126_) > 0.006, tqwt_TKEO_std_dec_12 (*a*_477_) > 0.005, std_10th_delta_delta (*a*_137_) ≤ 0.016, tqwt_kurtosisValue_dec_15 (*a*_732_) ≤ 4.112
23	0	3	3	0.000	Single-label	DT	std_delta_delta_log_energy (*a*_126_) > 0.006, tqwt_TKEO_std_dec_12 (*a*_477_) > 0.005, std_10th_delta_delta (*a*_137_) ≤ 0.016, tqwt_kurtosisValue_dec_15 (*a*_732_) > 4.112
25	6	0	6	0.000	Single-label	DT	std_delta_delta_log_energy (*a*_126_) > 0.006, tqwt_TKEO_std_dec_12 (*a*_477_) > 0.005, std_10th_delta_delta (*a*_137_) > 0.016, tqwt_stdValue_dec_19 (*a*_592_) ≤ 0.143
26	0	13	13	0.000	Single-label	DT	std_delta_delta_log_energy (*a*_126_) > 0.006, tqwt_TKEO_std_dec_12 (*a*_477_) > 0.005, std_10th_delta_delta (*a*_137_) > 0.016, tqwt_stdValue_dec_19 (*a*_592_) > 0.143

**Figure 7:**
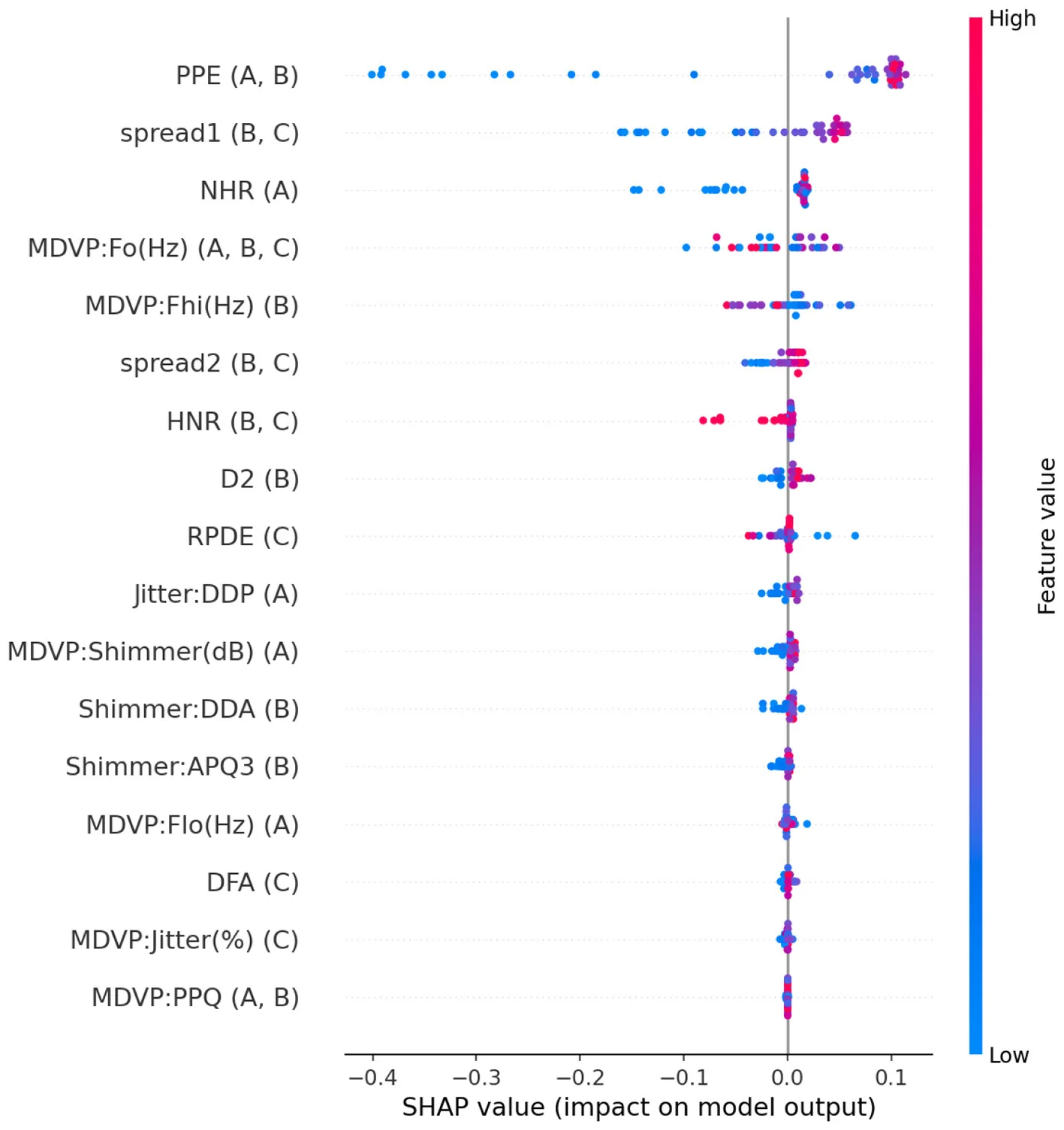
Beeswarm Plot for *D*_1_ dataset.

**Figure 8:**
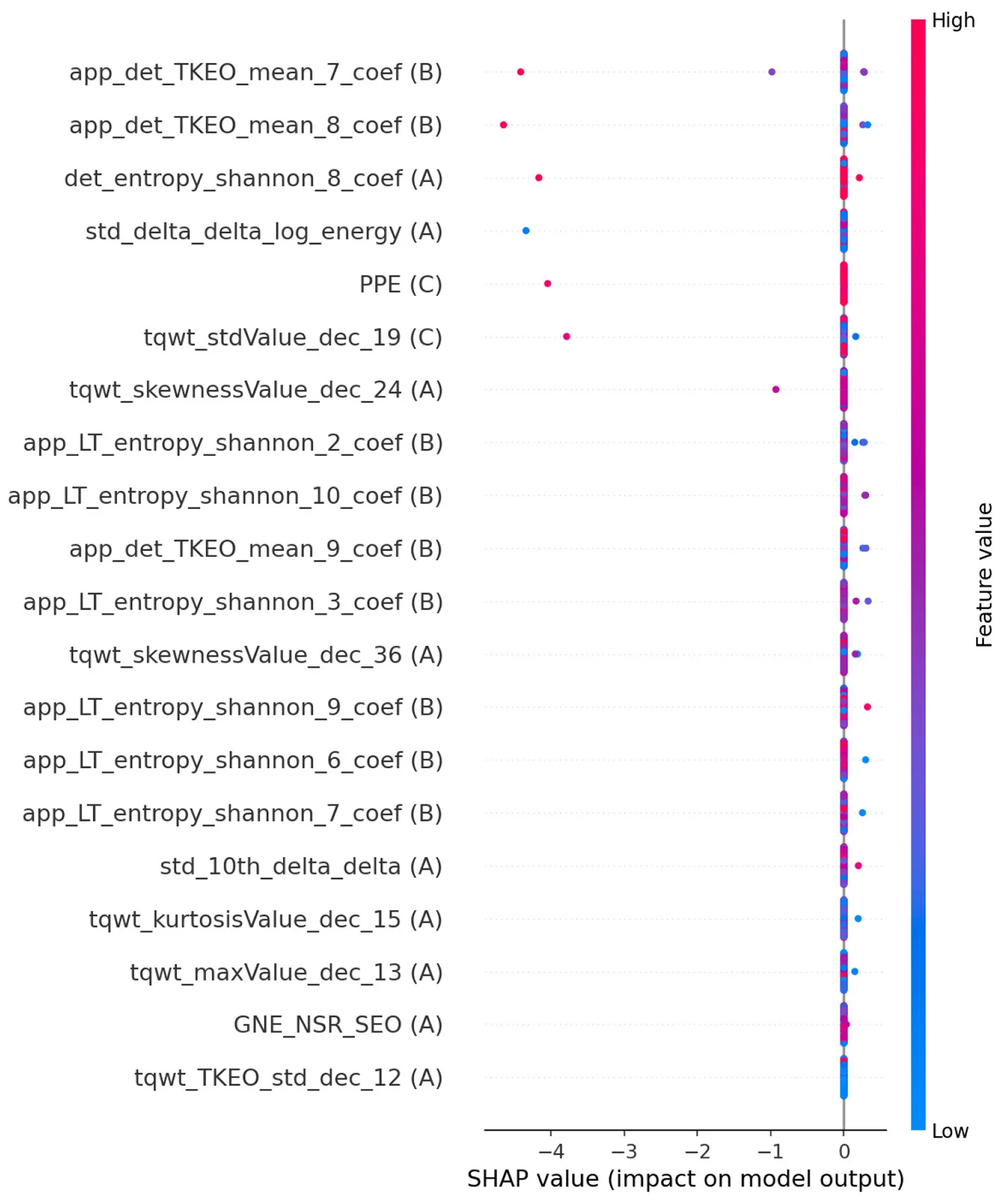
Beeswarm Plot for *D*_2_ dataset.

The proposed framework achieved a 96.98% accuracy on the *bagging* classifier with *ROS* balancing on Dataset *D*_1_ and 92.17% accuracy on the Gaussian process (*GP*) with *ROS* on Dataset *D*_2_ (Table 4). In comparison, the baseline Support Vector Machine (*SVM*) model on *D*_1_ achieved a recall of 97.28% but yielded a specificity of 45.83% (Table 2). Similarly, on Dataset *D*_2_, the *SVM* classifier achieved the recall of 97.70% but yielded a specificity of 43.75% (Table 2). Whereas after balancing, on the *D*_1_ dataset, *MLP* with the *SMOTE* balancing method's specificity resulted in 85.42%, while the corresponding classification accuracy had dropped to 90.68% (Table 3). Similarly, on the *D*_2_ dataset, both Bagging and Gradient Boosting (*GB*) specificity resulted in 65.62%, while the corresponding classification accuracy dropped to 82.92% (Table 3).

One of the most important results is the significant improvement of the specificity. The improvement reaches up to 30 percentage points for *GNB*, *SVM* and *KNN* classifiers. At the same time, the framework maintained high sensitivity avoiding the traditional trade-off between detection of PD cases and minimizing false alarms. Overall, the results suggest that IFI-ML leads to a more balanced and robust diagnostic model than conventional classifiers or resampling-based methods used separately. The framework also improves interpretability by means of the DecisionTreeLeafAnalyzer, which provides a confidence-aware representation of terminal nodes, enabling clinicians to find regions associated with high and low confidence predictions. IFI-ML decreases false-negative rates to 3-7% in *D*_1_ and 4–11% in *D*_2_, and limits false-positive rates to 3–7% in *D*_1_ and 12–20% in *D*_2_ from a clinical standpoint. Such improvements increase the probability of earlier disease detection and decrease unnecessary follow-up evaluations. Finally, SHAP-based analysis confirms that model predictions are largely driven by established vocal biomarkers, especially PPE and spread1 shown in Figures 7, 8. This agreement of these results with known pathophysiological features of PD is supportive of both the validity and clinical relevance of the proposed framework.

### 3.1 Baseline performance and trade-offs

The baseline 10-fold cross-validation results on Datasets *D*_1_ and *D*_2_ across fifteen classifiers are presented in Table 2. On the *D*_1_ dataset, classifiers achieve recall (*TPR*) values greater than 90% with lower specificity (*TNR*). In particular, the baseline Support Vector Machine (*SVM*) has a recall of 97.28% but specificity of 45.83%. The classification accuracy of the Multi-Layer Perceptron (*MLP*) over *D*_1_ is 90.16%, precision is 93.79%, recall is 92.52%, specificity is 81.25% and Matthews Correlation Coefficient (*MCC*) is 0.7277.

Performance indicators drop for several classifiers on *D*_2_ dataset. The MLP classifier has accuracy of 82.68% and specificity of 58.85% on *D*_2_ (Table 2) compared to accuracy of 90.16% and specificity of 81.25% on *D*_1_. The eXtreme Gradient Boosting (*XGBoost*) model accuracy decreases from 89.16% on *D*_1_ to 84.79% on *D*_2_. The CatBoost model provides the best baseline metrics on *D*_2_ with an accuracy of 85.45%, AUC-ROC of 0.9546, MCC of 0.8821, and specificity of 56.77% (Table 2).

### 3.2 Effect of class balancing (SMOTE and ROS)

Table 3 presents a summary of performance indicators after applying Random Over-Sampling (*ROS*) and *SMOTE*. For the Dataset *D*_1_, the specificity of the K-Nearest Neighbors (*KNN*) classifier is improved from 45.83% (baseline, Table 2) to 85.42% in the *SMOTE* configuration, and the *MCC* is improved from 0.4540 to 0.6143. The Gaussian Naive Bayes (*GNB*) has a specificity of 83.33% compared to the baseline of 45.83% and an *MCC* value that increases from 0.3839 to 0.4078. For the *SVM* classifier with *SMOTE*, the specificity increases from 45.83% to 66.67% whereas the recall decreases from 97.28% to 81.63% (Table 3).

*KNN* specificity is 81.25% in the dataset *D*_2_ which is an improvement from a baseline of 45.83% and a false positive rate of 18.75% decreased from 54.17% (Table 3). By applying *SMOTE*, *GNB*?s specificity increased from 63.02% to 66.15%, while its overall accuracy decreased from 75.66% (baseline) to 72.35%, and its *MCC* decreased from 0.7419 to 0.4159. *SMOTE* balancing on *D*_2_ for *CatBoost* gives accuracy of 81.74% and *MCC* of 0.5199 compared to baseline values of 85.45% and 0.8821 (Table 2).

When comparing the two balancing methods for Dataset *D*_1_, *SMOTE* has higher specificity than *ROS* for *KNN* (85.42% vs. 83.33%), *Extra Trees* (77.08% vs. 62.50%) and *MLP* (85.42% vs. 79.17%). The overall accuracies of some classifiers with *ROS* configuration are better than *SMOTE* on Dataset *D*_2_, e.g., *Bagging* (85.44% vs. 82.93%) and *Random Forest* (82.80% vs. 82.53%) (Table 3).

### 3.3 IFI ML: resolving the trade off

The overall classification performance of the *IFI* − *ML* framework under stratified 10-fold cross-validation is reported in Table 4. On the *D*_1_ dataset, the proposed framework achieves accuracies between 94.87-96.98% and *MCC* values exceeding 0.89 across all fifteen evaluated classifiers. On the larger *D*_2_ dataset, *IFI* − *ML* yields accuracies between 84.47-92.73% and *MCC* values exceeding 0.66.

For Dataset *D*_1_ under the *IFI* − *ML* *ROS* configuration, the *Bagging* classifier achieves an accuracy of 96.98% with a specificity of 96.34% (Table 4), compared to baseline values of 83.92% and 60.42% (Table 2). For the *SVM* classifier on *D*_1_, the baseline recall of 97.28% and specificity of 45.83% (Table 2) are recorded under *IFI* − *ML* *SMOTE* at 95.90% recall and 95.29% specificity, with an overall accuracy of 96.43% (Table 4). For the KNN classifier on *D*_1_, baseline specificity is 45.83% (Table 2) and *SMOTE*-only specificity is 85.42% (Table 3); under *IFI* − *ML* *SMOTE*, *KNN* specificity reaches 94.23% with an accuracy of 95.88% (Table 4).

On the high-dimensional *D*_2_ dataset, the baseline *CatBoost* model achieves a specificity of 56.77% (Table 2), while *IFI* − *ML* *GP* (under *ROS*) achieves an accuracy of 92.17%, a specificity of 84.46%, and a recall of 96.42% (Table 4). For the *KNN* model on *D*_2_, standard *SMOTE* balancing yields a specificity of 81.25% and a recall of 64.89% (Table 3), whereas *IFI* − *ML* *SMOTE* maintains a specificity of 82.03% and yields a recall of 95.40%, with an *MCC* of 0.7796 (Table 4). For the *QDA* model on *D*_2_, the baseline specificity of 34.90% (Table 2) and *SMOTE*-balanced *MCC* of 0.4159 (Table 3) are recorded under *IFI* − *ML* *SMOTE* at 75.83% specificity, 91.26% accuracy, and an *MCC* of 0.7253 (Table 4).

### 3.4 Interpretability and decision support

The structural partitioning of the feature space is governed by the adaptive Gini thresholds calculated via Equation 1, yielding τ_*D*1_ ≈ 0.28 for Dataset *D*_1_ and τ_*D*2_ ≈ 0.25 for Dataset *D*_2_. The resulting partitioning trees are visualized in Figure 5 (for *D*_1_) and Figure 6 (for *D*_2_).

The terminal node summaries reported in Table 5 identify single-label pure terminal nodes (Gini = 0.00), including Nodes 13 (98 Parkinson's samples) and 4 (6 Healthy samples) on *D*_1_, and Nodes 3 (20 Parkinson's samples) and 22 (31 Healthy samples) on *D*_2_.

Multi-label terminal nodes are partitioned into non-sensitive and sensitive regions using the calculated adaptive thresholds:

- Dataset *D*_1_ Non-Sensitive Nodes (Gini ≤ τ_*D*1_): Includes Node 6 (Gini = 0.067, 29 samples) and Node 11 (Gini = 0.157, 35 samples) (Table 5). Within these partitions, SVM specificity is recorded at 95.29%, KNN specificity at 96.34%, and *RF* specificity at 95.29% on Dataset *D*_1_ (Table 4).
- Dataset *D*_2_ Non-Sensitive Nodes (Gini ≤ τ_*D*2_): Includes Node 12 (Gini = 0.057, 68 samples) and Node 18 (Gini = 0.097, 372 samples) (Table 5). Within these partitions, the MLP model achieves 91.06% accuracy and 83.22% specificity on Dataset *D*_2_ (Table 4).
- Dataset *D*_1_ Sensitive Nodes (Gini > τ_*D*1_): Includes Node 10 (Gini = 0.408, 14 samples) and Node 14 (Gini = 0.500, 2 samples) (Table 5).
- Dataset *D*_2_ Sensitive Nodes (Gini > τ_*D*2_): Includes Node 13 (Gini = 0.480, 10 samples) and Node 17 (Gini = 0.356, 207 samples) (Table 5).

The sensitive boundary nodes are bypassed by the ML classifiers and routed directly to the gradient-descent-optimized Sugeno FIS pathway for local inference (Algorithm 1, lines 18–23).

### 3.5 Robustness, generalizability, and variance control

The performance stability of the proposed framework is compared against global resampling methods in Figure 9. On the high-dimensional Dataset *D*_2_, standard *SMOTE* balancing reduces the accuracy of the baseline *CatBoost* model to 81.74% with a corresponding *MCC* of 0.5199 (Table 3). Under the *IFI* − *ML* configuration, the same CatBoost variant achieves an accuracy of 86.85% and an *MCC* of 0.6944 (Table 4).

**Figure 9:**
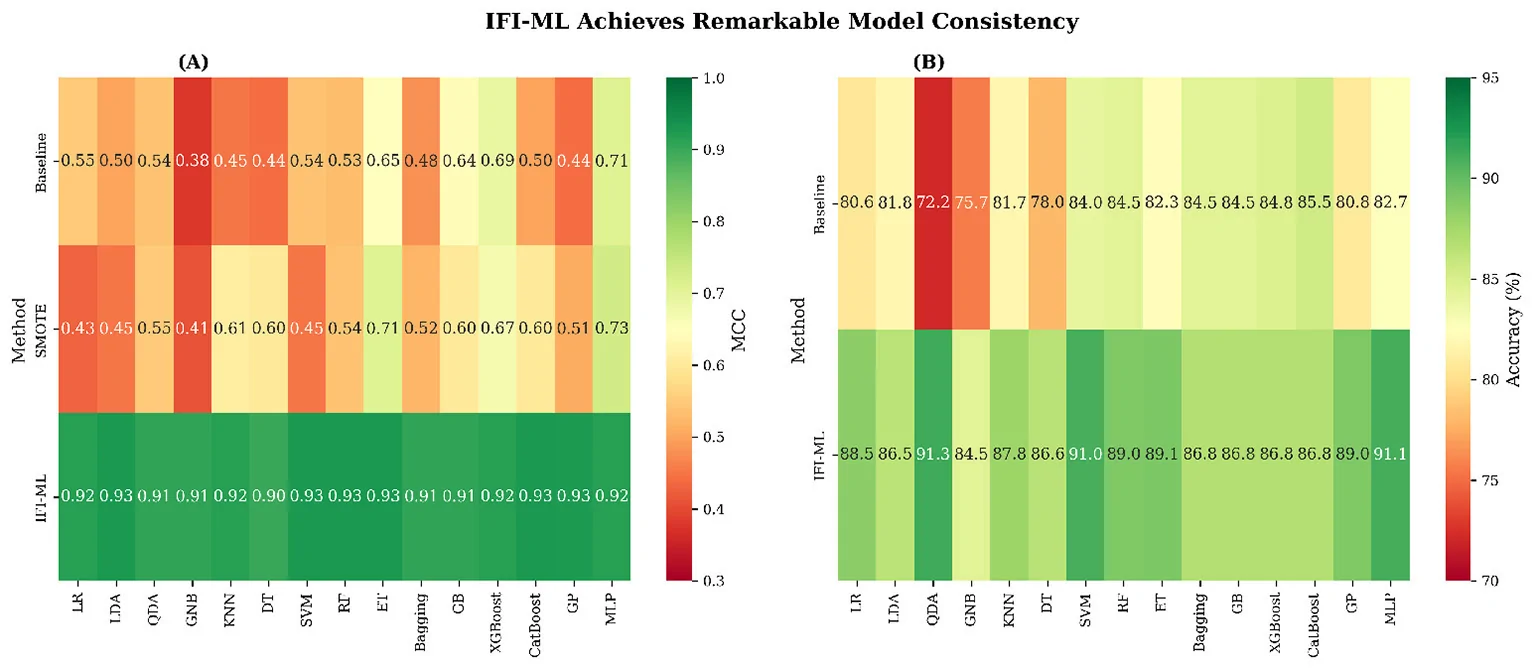
IFI-ML model consistency. **(A)** Dataset 1: MCC progression. **(B)** Dataset 2: Accuracy standardization.

On Dataset *D*_1_, standard *SMOTE* balancing yields an *SVM* specificity of 66.67% but reduces *SVM* recall from 97.28% (baseline, Table 2) to 81.63% (Table 3). The *IFI* − *ML* *SMOTE* framework yields an *SVM* recall of 95.90% and a specificity of 95.29% (Table 4). For the *GNB* classifier, *SMOTE* on *D*_2_ yields a specificity of 66.15% (Table 3), which rises to 83.71% under *IFI* − *ML* (Table 4). Across fourteen of the fifteen evaluated classifiers, *IFI* − *ML* maintains *MCC* values above 0.90 on Dataset *D*_1_ and above 0.77 on Dataset *D*_2_ (Table 4).

### 3.6 IFI-ML model interpretation

The beeswarm plot indicates that the features related to signal complexity and vocal instability, specifically pitch period entropy (PPE), spread1, NHR, MDVP:Fo(Hz), MDVP:Fhi(Hz), and spread2, have the highest absolute SHAP values on PD *D*_1_, which suggests that these features dominate global predictions on the IFI-ML model. High PPE and spread1 correlate with increased aperiodicity and loss of fine motor control in phonation, pushing predictions toward the Parkinson's class (right-shifting red points), whereas lower values indicate a healthy classification. High noise (high NHR, low HNR) is a strong PD indicator on multi-label non-sensitive and sensitive clusters. Lower average pitch (MDVP:Fo(Hz)) is often associated with PD. The color bar on the right is labeled “High” (red) to “Low” (blue), confirming high values in red push toward PD in many cases.

In PD patients, voice tends to show more instability (higher jitter/shimmer, more noise, non-linear irregularities), so high values in those features drive positive SHAP (toward PD prediction). Jitter- and shimmer-related measures such as Jitter:DDP, MDVP:Shimmer, and Shimmer:DDA show similarly interpretable trends, reflecting abnormal cycle-to-cycle frequency and amplitude perturbations commonly observed in PD voices. Non-linear dynamical features (*D*_2_, RPDE) and noise metrics (NHR) also contribute meaningfully, but with more moderate effect sizes. Overall, the SHAP distribution emphasizes that the IFI-ML's decisions align closely with established pathophysiological markers of dysphonia in Parkinson's disease, providing a transparent mapping between feature values and model influence for each subject. Comparison of the feature importance across the three feature sets based on the mean absolute SHAP values. The SHAP features of feature set A include Information Gain (IG) at depth=3, feature set B includes the Bagging classifier model, and feature set C includes FIS inference rules, which were combined into a unique list of 17 features. These 17 features include the classification of healthy individuals and Parkinson's, such as MDVP:Shimmer(dB), *D*_2_, Shimmer:APQ3, Shimmer:DDA, MDVP:PPQ, spread1, MDVP:Fhi(Hz), spread2, PPE, MDVP:Jitter(%), MDVP:Fo(Hz), MDVP:Flo(Hz), HNR, DFA, Jitter:DDP, RPDE, and NHR.

- Feature Set A (Single-Label): The information gain method on entire *D*_1_ samples includes features such as PPE, NHR, MDVP:Fo(Hz), Jitter:DDP, MDVP:Shimmer(dB), MDVP:Flo(Hz), and MDVP:PPQ.
- Feature Set B (Non-sensitive): The Bagging-ROS method on multi-label non-sensitve *D*_1_ includes features such as PPE, spread1, MDVP:Fo(Hz), MDVP:Fhi(Hz), spread2, HNR, *D*_2_, Shimmer:DDA, Shimmer:APQ5, and MDVP:PPQ.
- Feature Set C (Sensitive): The FIS-Rule on *D*_1_ multi-label sensitive samples includes features such as spread1, MDVP:Fo(Hz), spread2, HNR, RPDE, DFA, and MDVP:Jitter(%).

The above features of methods shown in Figure 7 appear to indicate that PPE and spread1 consistently play the significant roles across different sets, along with NHR and MDVP:Fo(Hz) features frequently. This finding suggests these features play a significant role in predicting the target variable.

On the other hand, the beeswarm plot for Sakar Parkinson's voice dataset *D*_2_ (combined features) shown in Figure 8 indicates that features capture vocal signal complexity, non-linear dynamics, and energy fluctuations. The analysis derives from three distinct feature selection approaches, where set A emphasizes information-theoretic aspects, set B non-sensitive features from performing QDA models, and set C sensitive features from fuzzy inference rule-based selection. The top features driving the combined model include a combination dominated by sets A and B, with PPE as the leading feature from set C. Here the feature dominance is unique on three sets in classifying the healthy and Parkinson's disease. Especially the approximate deterministic Teager-Kaiser Energy Operator (TKEO) mean coefficients (app_det_TKEO_mean_7_coef and app_det_TKEO_mean_8_coef) from feature set B, Shannon entropy (det_entropy_shannon_8_coef) and standard deviation of delta-delta log energy (std_delta_delta_log_energy) from set A, and PPE from set C exhibit the highest absolute SHAP values. These features dominate global predictions in the IFI-ML model, which achieves a mean cross-validated improved accuracy of around 8% for PD detection (85 to 92.7%).

Set B (non-sensitive) features have the greatest overall impact, followed by set A. Set C (sensitive) has a smaller but still significant contribution. Lower-ranked features like LT entropy coefficients, GNE_NSR_SEO, and tqwt skewness/kurtosis have weaker directional effects and narrower spreads. Use of mean absolute SHAP values to compare feature importance in the three feature sets. The distribution shows that high feature values (red points) cluster toward more negative SHAP values (left side of the plot), affecting model output more. Blue points with low feature values are closer to zero (right side). Higher values of these complexity- and instability-related features reduce model output, resulting in healthier class predictions. Lower values of these features have a less negative or near-zero impact, making Parkinson's predictions more accurate. The directionality is consistent across most of the highest-ranked features, but the effects vary. Set B's app_det_TKEO_mean coefficients are the most robust, followed by set A's entropy and log-energy variability. Moderate PPE effect. IFI-ML uses 25 features: 13 for Set A, 10 for Set B, and 2 for Set C.

- Feature set A (Single label): Emphasizes non-linear and energy-related measures on dataset *D*_2_ single label partitions primarily from det_entropy_shannon_8_coef, std_delta_delta_log_energy, tqwt_skewnessValue_dec 24 and 36, std_10th_delta_delta, tqwt_kurtosisValue_dec_15, tqwt_maxValue_dec_13, GNE_NSR_SEO, and tqwt_TKEO_std_dec_12. The other measures also include mean_MFCC_8th_coef, tqwt_energy_dec_6, tqwt_TKEO_std_dec 15, and 19.
- Feature set B (Non-sensitive): The QDA model uses the dominated features from Multi-label non-sensitive partitions by TKEO coefficients such as app_det_TKEO_mean_coef 7, 8, 9 and by entropy coefficients such as app_LT_entropy_shannon 2, 10, 3, 9, 6, 7 and 8.
- Feature set C (Sensitive): The FIS rule focused on established dysphonia markers from Multi-label sensitive or uncertain partitions such as PPE and tqwt_stdValue_dec_19.

The SHAP distribution explains the IFI-ML model's use of advanced signal complexity and energy features to distinguish Parkinson's and healthy voices. The observed negative directionality suggests dataset- or component-specific reduced irregularity or energy in PD phonation. Even though the directional influence differs from classical low-dimensional feature sets, this transparency helps validate the model's physiologically plausible speech alterations-based decisions.

## 4 Discussion

### 4.1 Methodological innovations

Entropy-based clustering and fuzzy inference integration of IFI-ML makes Parkinson's disease prediction more consistent across different types of data regions (Figures 3, 10). This setup configuration lets the framework handle both global distributional structure and localized uncertainty in the same pipeline. Compared to baseline methods, the Gini-based clusters make adaptive sensitivity (Figure 9) in the IFI-ML framework, which makes the performance of the system more stable. The framework is different from single-stage or single-model approaches since the framework deploys both machine learning classifiers and fuzzy logic. Moreover, this framework makes the system work efficiently with unclear or complex trials on the edge of the boundary (Figures 3, 9). These methodological components facilitate structured partitioning, context-sensitive processing, and consistent predictive performance across datasets.

**Figure 10:**
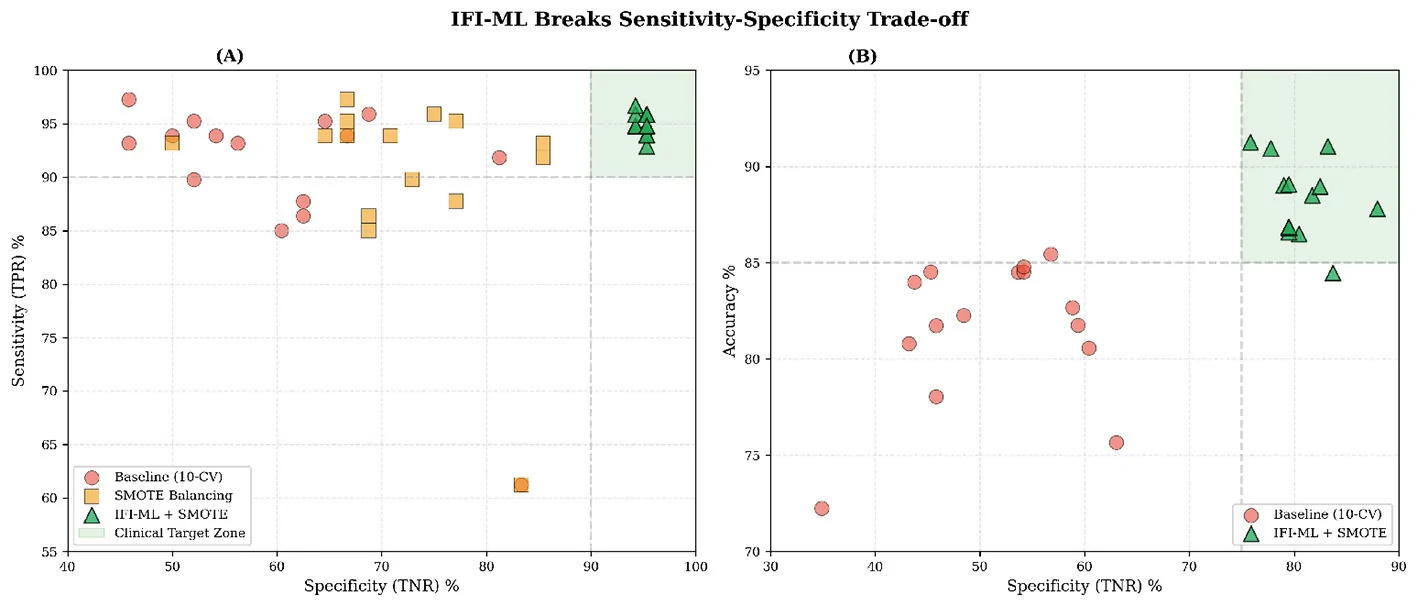
IFI-ML trade-off breakthrough. **(A)** Dataset 1: Trade-off resolution. **(B)** Dataset 2: Performance ceiling breakthough.

### 4.2 Clinical significance

IFI-ML reduces false negative rates to 3–7% and attains specificity of 94–95% on the dataset *D*_1_ and 75–88% on the dataset *D*_2_, supporting early Parkinson's detection while limiting false positives shown in Figures 3B, D. These gains remain consistent across model types and balancing techniques, as shown in Figures 9, 10. Earlier cross-validation and balancing methods alone exhibit higher false negative rates and inconsistent specificity, which can delay clinical interventions and increase unnecessary diagnostic procedures. IFI-ML integrates data partitioning and fuzzy inference to standardize predictive outcomes, enabling implementation flexibility based on computational resources and interpretability needs while minimizing diagnostic errors and workflow disruptions.

The results in Table 4 indicate that the performance gains achieved by IFI-ML extend beyond conventional class-distribution correction, reflecting a substantial improvement in decision-boundary modeling in Figure 4. On the *D*_1_ dataset, IFI-ML models converged to high specificity (94.23-96.34%) and recall (92.89-96.72%), whereas SMOTE-balanced models exhibited greater variability and a residual sensitivity-specificity trade-off. For instance, while SMOTE improved SVM specificity from a baseline of 45.83 to 66.67% at the cost of recall dropping to 81.63%, IFI-ML achieved 95.29% specificity and 95.90% recall establishing a highly balanced operating point. Moreover, these gains suggest that entropy-guided partitioning successfully reduces local class overlap, enabling learners to optimize clearer decision surfaces while the fuzzy inference component resolves ambiguous boundary samples.

This architectural advantage is particularly evident for the heterogeneous *D*_2_ dataset, for which the robustness of global resampling methods is limited by high dimensional feature spaces. Whereas KNN with SMOTE increased to 81.25% specificity but decreased to 64.89% recall, the IFI-ML configuration kept specificity to 82.03% but increased to 95.40% recall and MCC to 0.7796. Similar robust improvements were observed over CatBoost configurations, demonstrating that rather than redistributing classification errors, IFI-ML isolates high-impurity samples for processing through the Sugeno FIS instead of relying on unstable synthetic generation. Clinically, this uncertainty-aware framework facilitates a trustworthy decision-support layer where low false-negative rates (3-11%) ensure early Parkinson's Disease detection, and significantly reduced false-positive rates (3.66-24%) optimize healthcare resource allocation by minimizing redundant diagnostics.

### 4.3 Comparison with existing approaches

Tables 6, 7 show that this study's IFI-ML classification model had performed relatively well. Existing methods prioritize accuracy through resampling (SMOTE/ROS) or feature engineering, but boundary uncertainty, false positives/negatives, and interpretability in imbalanced medical data are the primary issues (Zhang et al., 2023; Salmi et al., 2024). From signal processing, the classification of Parkinson's Disease (PD) has evolved into more complex hybrid systems (Table 8). The existing works can be categorized into three main methodologies:

1. Feature-Centric Approaches: Early works by Little et al. (2009) and Sakar et al. (2019) aimed at extracting robust acoustic features (e.g., TQWT, MFCC) to improve the baseline accuracy. However, these approaches in imbalanced datasets are impacted by high-dimensional redundancy and a significant “false positive burden.”
2. Global Resampling and ML: Most of the research work applies global resampling techniques along with standard ML models. Dhar (2022) achieved 95.65% accuracy on the Parkinson dataset *D*_1_ by using an optimized *LightGBM* model. However, these approaches usually inflate sensitivity and specificity performance.
3. Fuzzy and Hybrid Systems: Chen et al. (2013) focused on Fuzzy KNN to deal with the uncertainty and obtained better performance on uncertain cases, but without a systematic method to separate obvious data from boundary samples.

**Table 6:** Comparative performance on Dataset *D*_1_ (UCI Parkinson voice, 10-fold cross-validation), including the proposed IFI-ML.

Study	Model/technique	Feature selection	Accuracy
Karapinar Senturk (2020)	CART	Feature importance	90.76
Karapinar Senturk (2020)	ANN	RFE	91.54
Karapinar Senturk (2020)	SVM	RFE	93.84
Chen et al. (2013)	FKNN	PCA	96.07
Chen et al. (2013)	SVM (RBF)	PCA	93.52
Chen et al. (2013)	SVM (Linear)	PCA	86.27
Ismail Cantürk and Karabiber (2016)	KNN (k=3)	LASSO	68.75
Ismail Cantürk and Karabiber (2016)	MLP (20 neurons)	Relief	68.94
Avuçlu and Elen (2020)	SVM	–	88.72
Avuçlu and Elen (2020)	KNN	–	85.64
Avuçlu and Elen (2020)	Random Forest	–	85.13
Dhar (2022)	XGBoost	MI–AE	91.21
Dhar (2022)	CatBoost	MI–AE	91.89
Dhar (2022)	LightGBM	MI–AE	92.34
Dhar (2022)	MI–AE–GOLGBM	MI–AE–GO	95.65
This study	IFI-ML	IG-clustering	96.98

This table includes only *D*_1_ studies explicitly reporting 10-fold CV in the source article.

**Table 7:** Comparative performance on Dataset *D*_2_ (UCI Parkinson voice, 10-fold cross-validation), including the proposed IFI-ML.

Study	Model/technique	Feature selection	Accuracy
Ariss and Hu (2023)	ResNet50	TQWT	90.7
Dhar (2022)	LightGBM	MI	87.69
Kuresan et al. (2021)	LR	Descriptive analysis	76.78
Kuresan et al. (2021)	LR	Factor analysis	78.23
Tuncer et al. (2020)	KNN	Relief + MAMT + SVD	92.46
Xiong and Lu (2020)	GBM/LR/SVM/NB/RF	AGWO	81–88
Solana-Lavalle and Rosas-Romero (2021)	RF	Wrapper (8–20)	92.2
Solana-Lavalle and Rosas-Romero (2021)	MLP	Wrapper (8–20)	86.64
Solana-Lavalle and Rosas-Romero (2021)	KNN	Wrapper (8–20)	85.58
Mostafa et al. (2019)	NN	MFEA	89.34
This study	IFI-ML	IG-clustering	92.7

This table includes only *D*_2_ studies explicitly reporting 10-fold CV in the source article.

**Table 8:** Summary of the core approaches.

Methodology	Core approach	Key advantages	Limitations
Traditional ML & Signal processing (Little et al., 2009; Sakar et al., 2013, 2018, 2019)	Extracting acoustic features and applying ML	High baseline accuracy on structured data.	High sensitivity to noisy features and data imbalance.
Global resampling (SMOTE/ROS) (Mei et al., 2021; Mahmud et al., 2021; Dhar, 2022)	Balancing for whole datasets.	Effectively handles minority class samples.	Fails to resolve boundary uncertainty; induces sensitivity-specificity trade-off.
SOTA ensembles (Zhang et al., 2023; Salmi et al., 2024; Egbo et al., 2025)	Global optimization & ensembles.	Optimization and explainability.	Often monolithic; applies the same logic to both simple and difficult cases.
This study	IFI-ML	Separates sensitive-boundary cases for targeted fuzzy logic.	Requires initial entropy-based partitioning.

This table summarizes the methodology, core approach, key advantages and limitations.

While hybrid/ensemble approaches (Zhang et al., 2023; Salmi et al., 2024) are effective, imbalance bias, overfitting, and sensitivity-specificity trade-offs are common. Voice classification of Parkinson's disease (PD) on UCI datasets *D*_1_ (Little et al., 2009) and *D*_2_ (Sakar et al., 2019) has been extensively studied using machine learning. IFI-ML uses entropy-based clustering to separate sensitive and non-sensitive samples, optimized ML on non-sensitive clusters, and Sugeno/Type-2 FIS on sensitive clusters or uncertain boundaries to fill these gaps. This method improves clinical trust, robustness, and balance over baselines and prior art. This version prioritizes model selection and performance diversity, reduces redundancy, and maintains readability. Previous methods used feature selection, classifiers, and balancing under 10 cross validations to achieve *D*_1_ accuracies of 68–95%. Due to the high dimensional feature size of *D*_2_, the training complexity of the ML models gets higher, but selecting TQWT/MFCC features, wrapper selection, and ensemble applications achieve 77–92%. Existing methodology uses advanced optimization to improve results, but imbalance, noisy features, and interpretability limit clinical reliability.

Figures 3, 9 shows the comparative results indicating that IFI-ML outperforms traditional balancing methods across various model types and data conditions. Figure 9 illustrates that previous methodologies frequently exhibit trade-offs between sensitivity and specificity, constraining the optimization potential of the model. Moreover, the existing baseline methods remain susceptible to varying or noisy data distributions, resulting in disparate outcomes across different implementation contexts, as illustrated in Figures 3D, 9B. As illustrated in Figures 3, 10, the IFI-ML framework enhances result consistency and demonstrates greater stability across all tested scenarios in diverse Parkinson's datasets.

### 4.4 Impact on medical AI development

IFI-ML achieves clinically meaningful improvements by delivering above 90% sensitivity on *D*_1_ and 87–96% on *D*_2_ while maintaining a high specificity of above 94% on *D*_1_ and 75-88% on *D*_2_, as shown in Figure 9A. Furthermore, the accuracy remains consistent at 95–97% on *D*_1_ and 87–93% on *D*_2_ across diverse models and balancing methods shown in Figures 3, 10. Whereas the earlier benchmarking methods often produce unstable models and force trade-offs, leading to an increase in diagnostic errors. By combining entropy-based clustering with fuzzy inference, IFI-ML stabilizes outcomes across heterogeneous datasets and eliminates these trade-offs, supporting trustworthy Parkinson's prediction and facilitating clinical adoption in routine diagnostic workflows.

## 5 Conclusion

The IFI-ML framework establishes a new benchmark for non-invasive Parkinson's disease classification by exceeding the limitations of monolithic, one-size-fits-all diagnostic models. The primary innovation of IFI-ML lies in an uncertainty-aware, dual-track architecture that utilizes entropy-guided partitioning to isolate and specifically address localized boundary uncertainty. Empirical validation on fifteen classifiers shows the state-of-the-art performance with IFI-ML reaching an optimum accuracy of 97% and MCC of 0.9400 on dataset *D*_1_, while also retaining the robust 92.17% accuracy and 0.7700 MCC on the heterogeneous *D*_2_ dataset. These results are a statistically significant improvement over existing global resampling methods which all performed inadequately in high-dimensional feature spaces.

Clinically, this framework efficiently overcomes the pervasive sensitivity-specificity tension that has long limited the reliability of medical AI. By selectively modeling high-entropy clusters via Sugeno-type fuzzy inference, IFI-ML provides a robust, interpretable tool that mitigates the clinical burden of false positives (3.66–24%) while ensuring high diagnostic sensitivity for early-stage screening. This entropy-driven integration improves cross-dataset robustness and provides a physiologically acceptable decision-support layer for practitioners. Future work will include longitudinal validation of this framework using multi-institutional evaluation and multimodal integration of motor and cognitive biomarkers to further refine diagnostic precision in complex clinical populations.

## Data Availability

The original contributions presented in the study are included in the article/supplementary material, further inquiries can be directed to the corresponding author.
